# Comprehensive Analysis of the Potential Immune-Related Biomarker Transporter Associated With Antigen Processing 1 That Inhibits Metastasis and Invasion of Ovarian Cancer Cells

**DOI:** 10.3389/fmolb.2021.763958

**Published:** 2021-12-10

**Authors:** Xiaoxue Li, Shiyu Zeng, Yiling Ding, Yanting Nie, Mengyuan Yang

**Affiliations:** Department of Obstetrics and Gynecology, The Second Xiangya Hospital of Central South University, Changsha, China

**Keywords:** pan-cancer, ovarian cancer, cervical cancer, transporter associated with antigen processing 1, tumor-infiltrating immune cells, immune checkpoint, metastasis

## Abstract

Transporter associated with antigen processing 1 (TAP1) is a protein related immune regulation and plays a role in several malignant tumors. However, the effect of TAP1 on immune infiltration, immunotherapy, and metastasis in different cancers has not been reported till date. The cancer genome atlas database, the tumor immune estimation resource database, and the estimation of stromal and immune cells in malignant tumors using expression (ESTIMATE) algorithm were used to determine the correlation between TAP1 expression and the prognosis of a variety of cancers, immune infiltration, immune checkpoint genes, DNA methylation, and neoantigens. Various enrichment analyses were used to study the correlation between TAP1 and key transcription factors using the Kyoto encyclopedia of genes and genomes (KEGG) pathway in ovarian cancer. Immunological methods were used to evaluate the expression of TAP1 protein in ovarian and cervical cancer, and Kaplan–Meier analysis was used to analyze the prognostic value of TAP1. RNA interference (RNAi) was used to verify the effect of TAP1 on ovarian cancer. Compared with normal tissues, cancer tissues showed a significant increase in the expression of TAP1, and TAP1 expression was related to the poor prognosis of cancers such as ovarian cancer. The expression level of TAP1 was correlated with immune checkpoint genes, DNA methylation, tumor mutation burden, microsatellite instability, and neoantigens in various cancers. Our results showed that TAP1 was upregulated in ovarian cancer cell lines and was associated with poor prognosis. Further, we verified the expression of TAP1-related transcription factors (MEF2A and LEF1) and found that TAP1 was closely related to ovarian cancer metastasis *in vitro* and *in vivo*. These results indicated that TAP1 could be used as a biomarker for the diagnosis and prognosis of cancer and as a new therapeutic target.

## Introduction

Cancer is a disease with the highest mortality rate worldwide. The incidence and fatality rate of cancer is increasing gradually every year and poses a serious threat to the lives and health of the people (Sung et al., 2021). Prostate cancer, colorectal cancer, and melanoma are the most common tumors among men. Breast cancer, cervical cancer (CC), and ovarian cancer (OC) are very common among women (Miller et al., 2019). Diagnosis of OC in their early stage is very difficult, and <70% of the patients are at late stages when their condition is diagnosed as OC (Torre et al., 2018). Early diagnosis and appropriate treatment can effectively improve the prognosis of cancer patients. Recent studies on tumorigenesis, diagnosis, and therapy have shown the importance of tumor immunity and the role of anti-programmed death 1/programmed death ligand 1 (anti-PD-1/PDL-1) and polyadenosine diphosphate-ribose polymerase (PARP) inhibitors (Mateo et al., 2019; Puri and Shafique, 2020). Thus, tumor immunity has become an emerging and popular direction of tumor research.

Transporter associated with antigen processing 1 (TAP1) belongs to a class of proteins closely related to antigen processing and immune regulation. TAP1 is responsible for processing exogenous pathogenic microorganisms and then presenting them to immune cells, thereby functioning as a target for tumor cells (Meng et al., 2018). Recent studies have reported the function of TAP1 in some cancers, but the role of TAP1 in immune infiltration, immunotherapy, and metastasis across various cancers has not been reported thus far.

We used the bioinformatics methods to determine the function of TAP1 in predicting the prognosis, immune infiltration, and immune checkpoint genes in various cancers. Additionally, we analyzed the correlation between TAP1 expression and tumor mutation burden (TMB) and microsatellite instability (MSI) and tumor neoantigens. Then, we determined the relationship between TAP1, pivotal transcription factors (TFs), and biological pathways in OC through gene enrichment analysis. In addition, we examined the expression of TAP1 protein in OC and CC cell lines by using western blot assays and its role in tumor metastasis *in vivo* and vitro. TAP1 protein expression in OC and CC tissues was determined by immunohistochemical staining, while Kaplan–Meier (KM) analysis was used to determine prognostic prediction function of TAP1. Our results showed that TAP1 could play an important role in immunotherapy across various cancers, and we determined the expression of TAP1 in cell lines and tissues to explain its role in the prognosis of CC and OC.

## Materials and Methods

### Processing Public Sequencing Data

The sequencing data of TAP1 in various tumor specimens and cell lines was derived from the cancer genome atlas (TCGA) database and cancer cell line encyclopedia (CCLE) database (Barretina et al., 2012). We enrolled samples pertaining to 20 tumors from the TCGA database (Weinstein et al., 2013) (https://www.cancer.gov/tcga), which included 8,624 tissues (7,900 tumor tissues and 724 adjacent normal tissues). Considering that some tumors had few adjacent samples, which may cause errors in the analysis, we imported 6678 healthy tissue and organ samples from the genotype-tissue expression (GTEx) database (Carithers and Moore, 2015) (https://www.gtexportal.o-rg/home/). Tumors with missing data were not included in the calculation of the difference in gene expression between tumor and healthy tissues. The Kruskal-Wallis test was used to compare TAP1 expression differences between different tumors or cells. We used the combat function of the R software package “SVA” to remove the batch effect and normalized the expression of the rest samples. After that, Wilcoxon test was utilized to illustrate the expression of TAP1 in tumor tissues and the corresponding normal tissues. A value of *p* < 0.05 was considered to be statistically significant.

### The Analysis of TAP1 in Predicting the Prognosis of Cancer Patients

The survival data of 7,900 patients with cancer were derived from the TCGA database, which included data on overall survival (OS), disease-specific survival (DSS), disease-free survival (DFS), and progression-free survival (PFS). We used the KM survival analysis to determine the predictive value of TAP1 in various cancers, and the log-rank test was used to calculate the significance. The results were considered to be statistically significant when *p* < 0.05.

### Correlation Analysis Between Immune Infiltration and TAP1 Expression

The tumor immune estimation resource (TIMER) database (Li et al., 2020) (http://timer.cistrome.org/) used a method known as deconvolution, which applied sequencing data to calculate the abundance of tumor infiltrating immune cells (TIICs) in tumor tissues. The TIMER database was used to systematically analyze the TIICs in 32 kinds of cancers using 10,897 tissues from the TCGA. Using this database, we determined the correlation between the TAP1 gene expression and the abundance of TIICs (neutrophils, CD4^+^ T cells, B cells, CD8^+^ T cells, macrophages, and dendritic cells) in 32 tumors.

### Analysis of the Relationship Between Estimation of Stromal and Immune Cells in Malignant Tumors Using Expression Scores and TAP1 Expression Level

Estimation of stromal and immune cells in malignant tumors using expression (ESTIMATE) (https://bioinformatics.mdanderson.org/publicsoftware/estimate/) was an algorithm that applied sequencing profiles to infer the extent of immune infiltration in a tumor microenvironment, including stromal and immune cells. The sequencing profiles of 8,624 tissues from 20 types of cancers were used to estimate the proportion of immune and stromal cells in each tissue through limma packages. STROMAL score, IMMUNE score, and ESTIMATE score were three ESTIMATE scores which described the immune infiltration. Then, we integrated the profiles of TAP1 and ESTIMATE scores to calculate the correlations. The results were considered to be statistically significant when *p* < 0.05.

### The Correlation Between TAP1 Expression and TMB and MSI

TMB and MSI were the 2 metrics which could influence the treatment effect of tumor immunotherapy. The gene mutation profiles of 10,114 tissues of 33 cancers from the TCGA database were used to calculate gene variation in every tissue. The Spearman correlation test was used to determine the relationship between TAP1 and TMB using the fmsb package (https://CRAN.R-project.org/package=fmsb), and then, we drew the correlation radar map. A procedure similar to that for TMB scores was followed for the MSI scores.

### Correlation of TAP1 With Immune Checkpoint Genes and Methyltransferase

After analyzing the correlation of TAP1 with ESTIMATE scores and TIICs, we further investigated the relationship between TAP1 and immune checkpoint genes. We summarized 47 immune checkpoint genes mentioned in the literature, and then, we used the limma package to determine the relationship between TAP1 and the 47 genes in 33 tumors. The Spearman correlation test was applied to calculate the significant difference. We used the reshape2 package (http://www.jstatsoft.org/v21/i12/) to draw the correlation heat plot. DNA methylation was a form of DNA chemical modification which could alter genetic performance without changing the DNA sequence by 4 methyltransferases (DNMT1, DNMT2, DNMT3A, and DNMT3B). The relationship between TAP1 and the 4 methyltransferases was calculated using the method same as above.

### Gene Set Enrichment/Variation Analysis

To determine the signaling pathways and biological functions of TAP1 in OC, we divided the OC tissues into high-expression and low-expression group according to the level of expression of TAP1, which was determined using limma,org.Hs.eg.db, clusterProfiler (http://www.bioconductor.org/packages/release/bioc/html/clusterProfiler.html). We employed the enrich-plot package based on the c5.all.v7.1.symbols file to perform gene set enrichment analysis (GSEA) or gene set variation analysis (GSVA) on the sequencing data of the OC samples from the TCGA database. Then, we selected the top TFs and KEGG terms to draw the enrichment maps. The results were statistically significant when *p* < 0.05.

### Quantification of Mutation/Neoantigens Load

Neoantigen was an abnormal protein produced by somatic cell mutation. After being degraded into short peptides, it could be identified by antigen-presenting cells (APCs), which were mainly dendritic cells (DC cells). According to the tumor neoantigen defined by Rooney et al. (2015), we counted the mutations in each sample and annotated somatic mutations with variant effect predictor (VEP). We used the NetMHCpan (v2.4) algorithm to predict whether the tumor cells in 33 types of tumors would produce new antigens that were different from ordinary cells. The NetMHCpan software was used to predict the affinity between peptides and major histocompatibility complex (MHC) type I molecules. On the basis of the artificial neural network algorithm, the training set was constructed with a complex of binding affinity profiles and MHC-eluted ligand data.

### Immunohistochemistry and KM Analysis

We randomly selected 41 human OC specimens and 32 CC specimens from patients undergoing surgery during 2013–2015 after obtaining their consent; the specimens were collected from the department of pathology at The Second Xiangya Hospital of Central South University. The experiment was approved by the Human Ethics Committee of The Second Xiangya Hospital of Central South University. The average survival time of patients with OC in our 5-years analysis was 43 months, while the longest survival time was 89 months. The average survival time of patients with CC was 57 months, while the longest survival time was 85 months. The survival data of each patient was confirmed by phone or questionnaire. The tissue obtained from the patients was embedded in paraffin and slices were prepared. After the sections were deparaffinized and dehydrated, the Tris-EDTA buffer was used for antigen retrieval. Subsequent incubation with antibody and staining using diaminobenzidine (DAB) were performed according to the non-biotin detection kit (ZSGB-BIO, PV-9001, China). The method was similar to that described previously (Li et al., 2019).

### Western Blotting

We used radioimmunoprecipitation assay (RIPA) buffer to lyse the cell lines cultured in 6-cm dishes to prepare protein samples. Then, we centrifuged the samples at 12,000 g for 5 min at 4°C in a 1.5-ml tube and then transferred the cell extract to a new tube, added 5*sodium dodecyl sulfate (SDS) loading buffer and heated it for 5 min at 95°C. The electrophoresis conditions were 120 V, 50 min. After electrophoresis, a polyvinylidene difluoride (PVDF) membrane was used for protein transfer at conditions of 400 mA, 45 min. The PVDF membrane was blocked using 5% skim milk diluted with Tris-buffered saline Tween 20 (TBST). The membrane was incubated with the following primary antibodies TAP1 (1:3,000), MEF2A (1:2000, #12382-1-AP, Proteintech, United States), LEF1 (1:1000, #14972-1-AP, Proteintech), E-cadherin (1:10000, #20874-1-AP, Proteintech), vimentin (1:2000, #10366-1-AP, Proteintech), and beta actin (1:1000, # 20536-1-AP, Proteintech). After overnight incubation, the membrane was labeled using horseradish peroxide (HRP)-conjugated secondary antibody. Then, we washed the PVDF membrane and applied electrochemiluminescence (ECL) luminescent solution to develop the pictures.

### Small Interfering RNA Transfection

We designed 2 different sequences to knock down TAP1 mRNA in OC cell lines. The small interfering RNAs (siRNAs) (si-TAP1_01 and si-TAP1_02) and negative control siRNA (si-NC) were obtained from RiboBio (Guangzhou, China). The cells were seeded into 35-mm cell culture dishes at a density of 2 × 10^4^ cells/dish and transfected with siRNA using riboFECTTM CP (Guangzhou, China) after reaching 70% confluence. After 48 h of incubation, the efficiency of siRNAs was assessed using quantitative real-time polymerase chain reaction (qRT-PCR) and western blotting analysis. The target sequences of the TAP1 siRNAs in TAP1 were as follows: Si-TAP1_01, 5′-CGG​GAT​CTA​TAA​CAA​CAC​CAT-3′; Si-TAP1_02, 5′-CCG​TGT​GTA​CTT​ATC​CTG​GAT-3′.

### Cell Migration and Wound Healing Assays

Cell migration assays were conducted using transwell inserts with 8-μm pore filters (Corning, NY, United States). After transfecting the OC cell lines with TAP1 siRNAs or negative control siRNA, the cells were digested and resuspended in 200-μL serum-free medium, and then inoculated in the upper chamber of the transwell chamber in a 24-well plate. RPMI-1640 (Gibco, United States) containing 20% fetal bovine serum (FBS; Gibco) was added to the bottom well. After 24 h of incubation, the cells remaining on the surface of the upper chamber were removed, and the cells entering the lower chamber were fixed with 10% formalin and stained with crystal violet staining solution (Beyotime, Shanghai, China). Then, the stained cells were photographed at a 100× magnification. For the wound healing assays, the transfected cells were seeded into 12-well plates and cultured until almost overgrowth. Then, we created a wound by scratching the cells using 100-μL pipette tips. Cell migration was evaluated by measuring the gap area in multiple fields after 0 and 24 h at a magnification of 100×.

### Cell Proliferation Assays and Colony Formation Assays

In cell proliferation assays, we seeded SKOV3 and OVCAR3 in 96-well plates at a density of 1 × 10^4^/well and transfected with si-TAP1_01, si-TAP1_02 or si-NC for 24, 48, 72 or 96 h at 37°C with 5% CO_2_. Then 10 μL Cell Counting Kit-8 solution (CCK-8, #C0037, Beyotime, Shanghai, China) was added and incubated for 60 min. The absorbance of formazan produced was detected at 450 nm under microplate reader (Thermo Scientific, CA, United States). In colony formation assays, the transfected SKOV3 and OVCAR3 cells were seeded in 6-well plates at a density of 1×10^3^/well and kept for 14 days. Then the clonies were stained with crystal violet staining solution (Beyotime, Shanghai, China) and imaged.

### Xenograft Experiments

All animal experiments were approved by the Scientific Investigation Board of the Central South University (Changsha, China) and conducted in according to the National Institute of Health Guide for the Care and Use of Laboratory Animals. For *in vivo* tumor metastasis assays, eight female BALB/c nude mice (6 weeks old) were purchased from SJA Laboratory Animal (Changsha, China) and maintained in a specific pathogen-free facility. All nude mice were injected intraperitoneally (i.p.) with 5 × 10^6^ cells in 200 μL of phosphate-buffered saline (PBS). Mice were randomly selected for treatment with cholesterol conjugated si-TAP1_01 or si-NC (*n* = 4/group) provided by RIBOBIO (Guangzhou, China) and 25 nmol RNA in 0.1 ml PBS was injected i.p. twice a week. The body weight of mice were measured each week. Four weeks after inoculation the mice were sacrificed and visceral organs (liver, intestine, mesentery, kidney, ovary and diaphragm) were observed. Visible tumors were harvested for weight and western blotting assays.

### Statistical Analysis

We used the limma package to determine the expression level of TAP1 in tumors and the Wilcoxon test to calculate the significant difference. KM analysis was used to analyze the prognostic value of TAP1 using the log-rank test. Spearman method was applied to analyze the correlations between TAP1 and immune cells and immune genes. The R version 3.6.2 software (https://www.r-project.org/) was used for data analysis. *p* < 0.05 was considered statistically significant.

## Results

### Pan-Cancer Expression of TAP1

**FIGURE 1:**
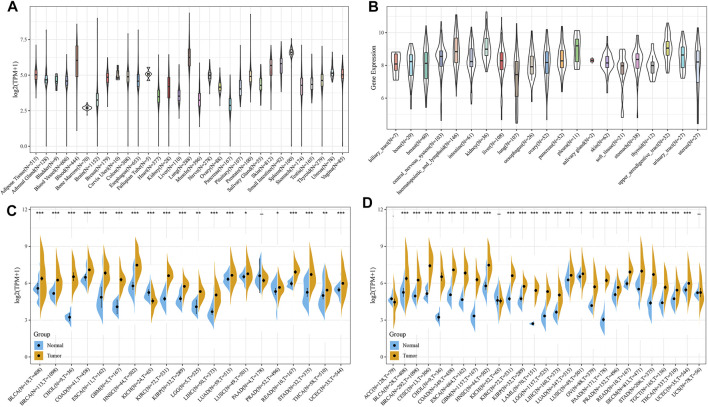
The expression of transporter associated with antigen processing 1 (TAP1) in pan-cancer. **(A)** TAP1 expression of 31 kinds of normal tissues downloaded from genotype-tissue expression (GTEx) database. **(B)** TAP1 expression in 21 kinds of cancer cell lines derived from the cancer cell line encyclopedia (CCLE). **(C)** TAP1 expression in tumor samples and the adjacent normal samples of 20 cancers derived from the TCGA. **(D)** TAP1 expression in tumor samples and normal samples of 27 cancers derived from the cancer genome atlas (TCGA) and GTEx database. *p < 0.05. **p < 0.01. ***p < 0.001.

Analysis of the sequencing data of TAP1 from 31 normal tissues derived from the GTEx dataset showed overexpression of TAP1 mRNA in many tissues, including blood, spleen, and lungs (Figure 1A). The expression profiles of 21 cancer cell lines derived from the CCLE dataset showed high expression levels of TAP1 in the kidney, pleura, and lymphoid cancer cell lines (Figure 1B). We collected the transcriptome sequencing data of 20 cancers vs. adjacent normal tissue from the TCGA dataset, consisting of 8,624 samples. The results showed that compared with normal tissues, tissues of various cancers, including head and neck squamous cell (HNSC), kidney renal clear cell carcinoma (KIRC), and stomach adenocarcinoma (STAD), showed increased levels of TAP1 expression (Figure 1C). To date, however, the TCGA database does not have sufficient data on TAP1 expression in different kinds of tumors, including OC and its adjacent tissues. Thus, we matched the normal samples obtained from GTEx with the corresponding samples in TCGA and analyzed the expression data of 27 cancers. Our results showed that TAP1 was upregulated in cervical squamous cell carcinoma (CESC), esophageal carcinoma (ESCA), OC, pancreatic adenocarcinoma (PAAD), acute myeloid leukemia (LAML), etc. (Figure 1D), indicating the role of TAP1 as a tumor marker across various cancers.

### The Predictive Ability of TAP1 in the Prognosis of Various Cancers

**FIGURE 2:**
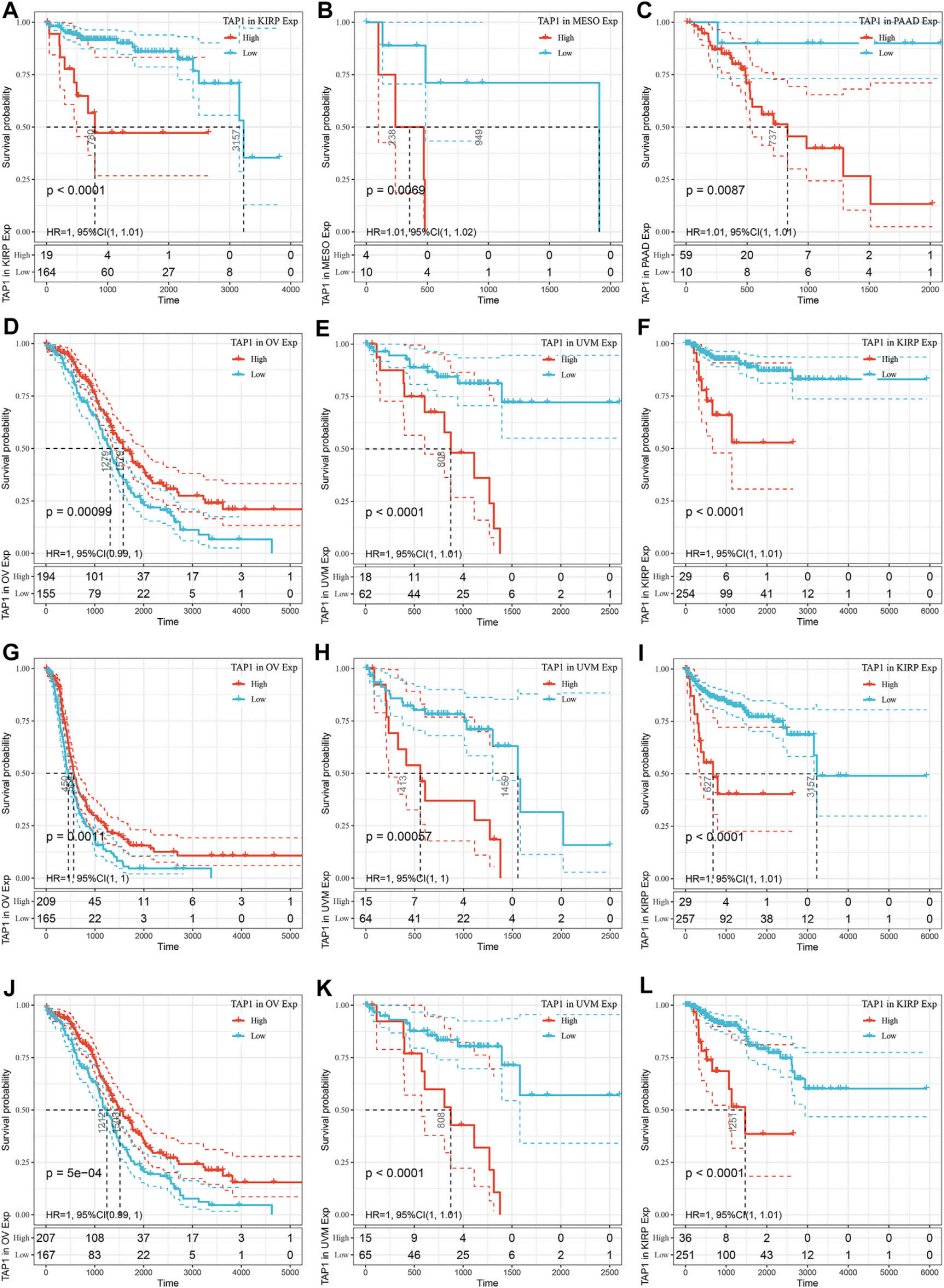
The prediction ability of transporter associated with antigen processing 1 (TAP1) in pan-cancer prognosis. **(A–C)** The top three cancers correlated with TAP1 in disease-free survival (DFS). **(D–F)** The top three cancers correlated with TAP1 in disease-specific survival (DSS). **(G–I)** The top three cancers correlated with TAP1 in progression-free survival (PFS). **(J–L)** The top three cancers correlated with TAP1 in Overall survival (OS).

To explore the ability of TAP1 in predicting the prognosis of various cancers, we used the KM analysis to calculate the relationship between TAP1 expression and survival status in 33 tumors using the patient information in the TCGA. The results showed that the TAP1 expression level was correlated with DSS (Figures 2D–F), PFS (Figures 2G–I), and OS (Figures 2J–L) in kidney renal papillary cell carcinoma (KIRP) (DSS: HR = 1.01, *p* < 0.0001; PFS: HR = 1.01, *p* < 0.0001; OS: HR = 1.01, *p* < 0.0001), uveal melanoma (UVM) (DSS: HR = 1.01, *p* < 0.0001; PFS: HR = 1.00, *p* = 0.00057; OS: HR = 1.00, *p* < 0.0001), and OC (DSS: HR = 1.01, *p* < 0.0001; PFS: HR = 1.00, *p* = 0.0011; OS: HR = 1.00, P = 5e-04). High levels of TAP1 expression were related with shorter DFS in KIRP (HR = 1.00, *p* < 0.0001), mesothelioma (MESO) (HR = 1.00, *p* = 0.0069), and PAAD (HR = 1.00, *p* = 0.0087) (Figures 2A–C). In addition, our results showed that TAP1 was associated with DSS, PFS, OS in different kinds of tumors such as brain lower grade glioma (LGG), skin cutaneous melanoma (SKCM), and bladder urothelial carcinoma (BLCA) (Supplementary Figures S1–S3). Thus, our results indicated further studies should be performed to establish the role of TAP1 as a prognostic marker in many cancers.

### The Correlation of TAP1 With Immune Infiltration in Multiple Cancers

**FIGURE 3:**
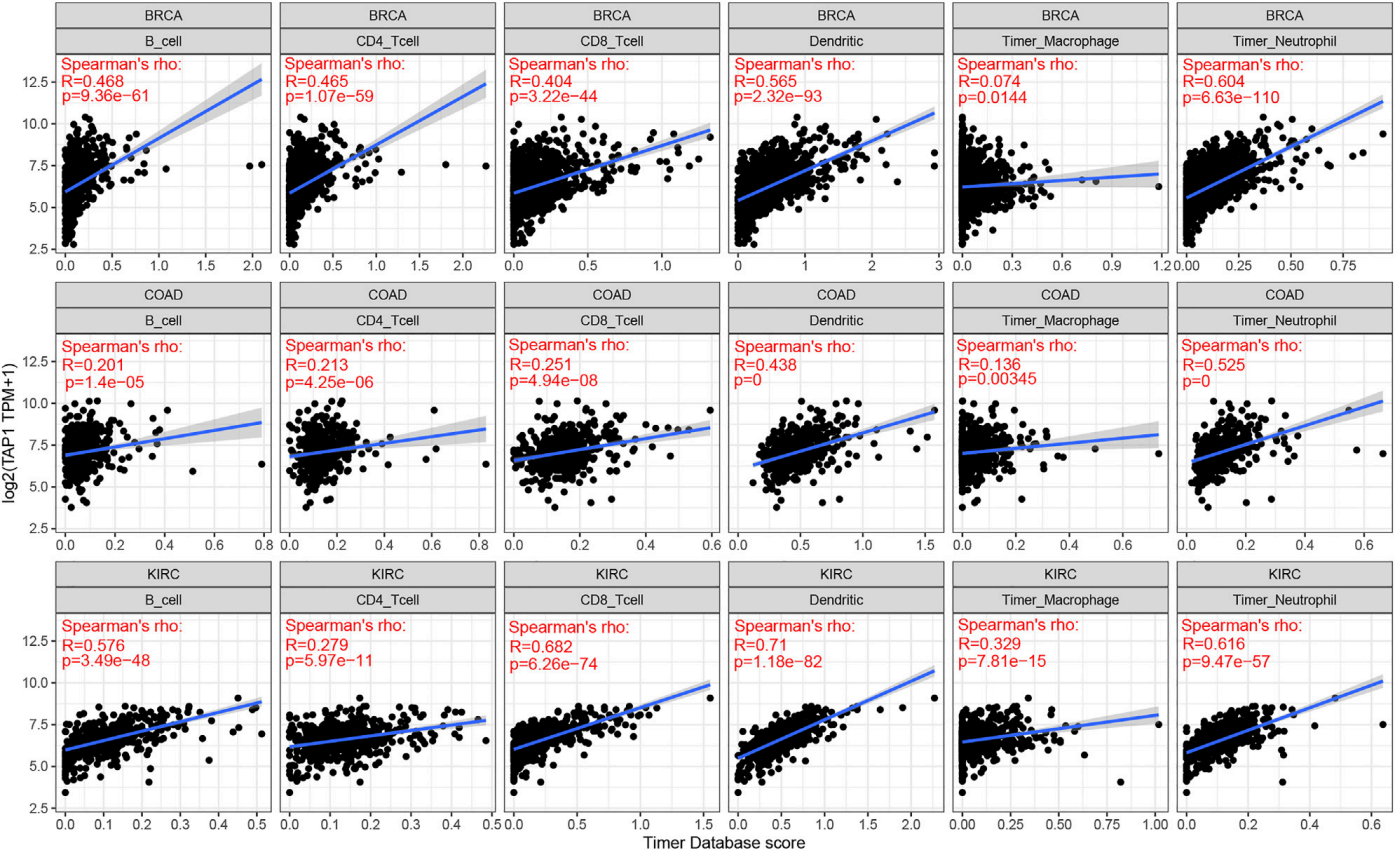
The correlation of transporter associated with antigen processing 1 (TAP1) with immune infiltration in multiple cancers. The top three cancers with the strongest correlation with 6 immune infiltration cells which included B cells, CD4+T cells, CD8+T cells, neutrophils, macrophages, and dendritic cells. The cancers were colon adenocarcinoma (COAD), kidney renal clear cell carcinoma (KIRC), and breast invasive carcinoma (BRCA), respectively.

**FIGURE 4:**
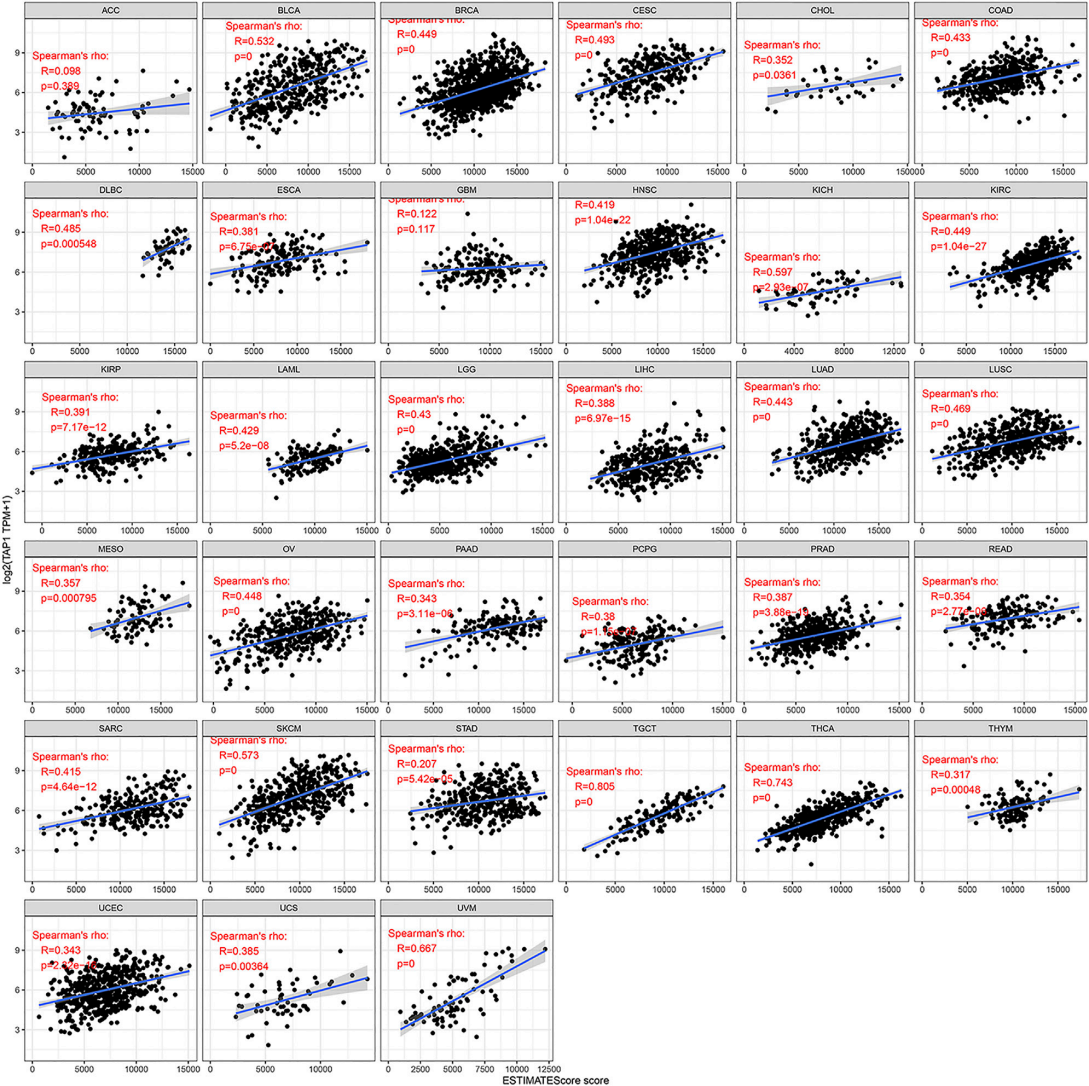
The relationship between TAP1 and ESTIMATE score in multiple cancers. The ESTIMATE score was used to determine the correlation of TAP1 with immune cells and stromal cells in 33 tumors. TAP1 was positively correlated with ESTIMATE scores in cervical squamous cell carcinoma (CESC), ovarian cancer (OC), uveal melanoma (UVM), testicular germ cell tumors (TGCT), and thyroid carcinoma (THCA) (*p* < 0.05).

TAP1 was an important regulator involved in immune response, while immune infiltration could influence the development and treatment effect of tumors. The TIMER database was used to analyze the correlation of TAP1 with TIICs in 27 tumors. Results of this analysis showed that in some tumors, TAP1 was significantly correlated with 6 TIICs, including B cells, CD4+T cells, CD8+T cells, neutrophils, macrophages, and dendritic cells. The top 3 tumors were colon adenocarcinoma (COAD), KIRC, and liver hepatocellular carcinoma (LIHC) (Figure 3). Additionally, TAP1 was positively related with B cells (R = 0.341, *p* = 1.99e-11), CD4+T cells (R = 0.419, *p* < 0.0001), CD8+T cells (R = 0.488, *p* < 0.0001), neutrophils (R = 0.616, *p* < 0.0001), macrophages (R = 0.137, *p* = 0.0001), and dendritic cells (R = 0.592, *p* < 0.0001) in OC. Similarly, in CESC, TAP1 was positively related with B cells (R = 0.240, *p* = 5.28e-08), CD4+T cells (R = 0.252, *p* = 1.12e-08), CD8+T cells (R = 0.515, *p* = 2.63e-35), neutrophils (R = 0.491, *p* = 9.4e-32), macrophages (R = 0.167, *p* = 0.0002), and dendritic cells (R = 0.511, *p* < 0.0001) (Supplementary Figure S4). The ESTIMATE algorithm was used to determine the correlation of TAP1 with immune cells and stromal cells in 33 tumors. The ESTIMATE algorithm included 3 immune-related scores: ESTIMATE score, STROMAL score, and IMMUNE score. The results demonstrated that the expression of TAP1 had a significant correlation with immune-related scores. TAP1 was positively correlated with ESTIMATE scores in CESC (R = 0.493, *p* < 0.0001), OC (R = 0.448, *p* < 0.0001), UVM (R = 0.667, *p* < 0.0001), testicular germ cell tumors (TGCT) (R = 0.805, *p* < 0.0001), and thyroid carcinoma (THCA) (R = 0.743, *p* < 0.0001), etc. (Figure 4). Furthermore, TAP1 was positively correlated with IMMUNE score and STROMAL score in different kinds of tumors, including OC and CESC (Supplementary Figures S5, S6).

### The Relationship Between TAP1 and Immune Checkpoint Therapy in Multiple Cancers

**FIGURE 5:**
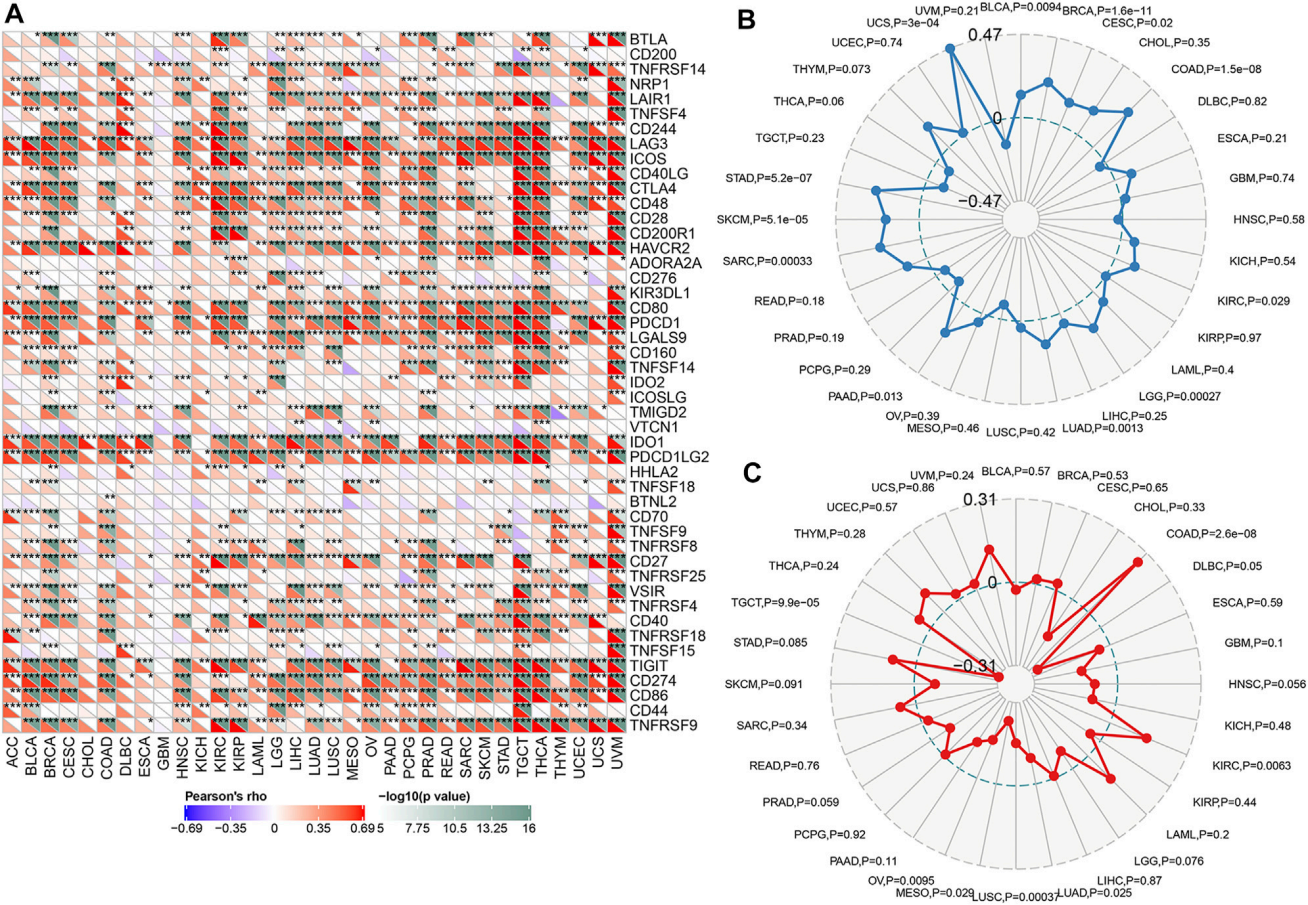
The relationship between transporter associated with antigen processing 1 (TAP1) and immune checkpoint therapy in multiple cancers. **(A)** The relationship between TAP1 and 47 immune checkpoint genes. Color green represented negative correlation with TAP1 and color red represented positive correlation with TAP1. **(B)** The correlation of TAP1 with tumor mutation burden (TMB). **(C)** The correlation of TAP1 with microsatellite instability (MSI). *p < 0.05. **p < 0.01. ***p < 0.001.

Immune checkpoints referred to a group of molecules present in immune cells that could participate in preventing the activation of the immune system. Tumor cells could inhibit the function of T cells by activating immune checkpoints, thereby achieving tumor immune escape. Typically, anti- PD-1, anti- PD-L1, and anti-cytotoxic T lymphocyte antigen 4(CTLA-4) therapy was administered in the treatment of tumors. We analyzed the expression of TAP1 with 47 immune checkpoint genes through the expression data in 33 tumors. The results demonstrated that TAP1 was expressed together with many immune checkpoint genes in various tumors. For example, in OC and CESC, TAP1 was positively correlated with CD274, TIGHT, CTLA4, and LAG3 (Figure 5A). These results indicated that TAP1 may affect immune therapy in tumor through immune checkpoint genes. TMB was an index that reflects the abundance of gene mutations in cancer. MSI was a phenomenon in which a new microsatellite allele appeared at a certain site in the tumor because of the insertion or deletion of a repeat unit. Both were important metrics correlated with the effect of immune checkpoint therapy such as PD-1 and PDL-1. The results indicated that the expression TAP1 was positively related to TMB in CESC, breast invasive carcinoma (BRCA), COAD, lung adenocarcinoma (LUAD), PAAD, sarcoma (SARC), SKCM, and STAD, whereas the highest score was observed for the correlation of TAP1 and COAD. Further, TAP1 was negatively correlated with TMB in BLCA (Figure 5B). In addition, TAP1 negatively correlated with MSI in OC, MESO, lung squamous cell carcinoma (LUSC), LUAD, diffuse large B-cell lymphoma (DLBC), and TGCT, while the correlation in TGCT had the strongest score. The expression of TAP1 was positively related to MSI in COAD and KIRC (Figure 5C). Overall, we found that TAP1 could possibly affect the tumor immunotherapy.

### The Correlation of TAP1 With Neoantigens and Methyltransferases Across Various Cancers

**FIGURE 6:**
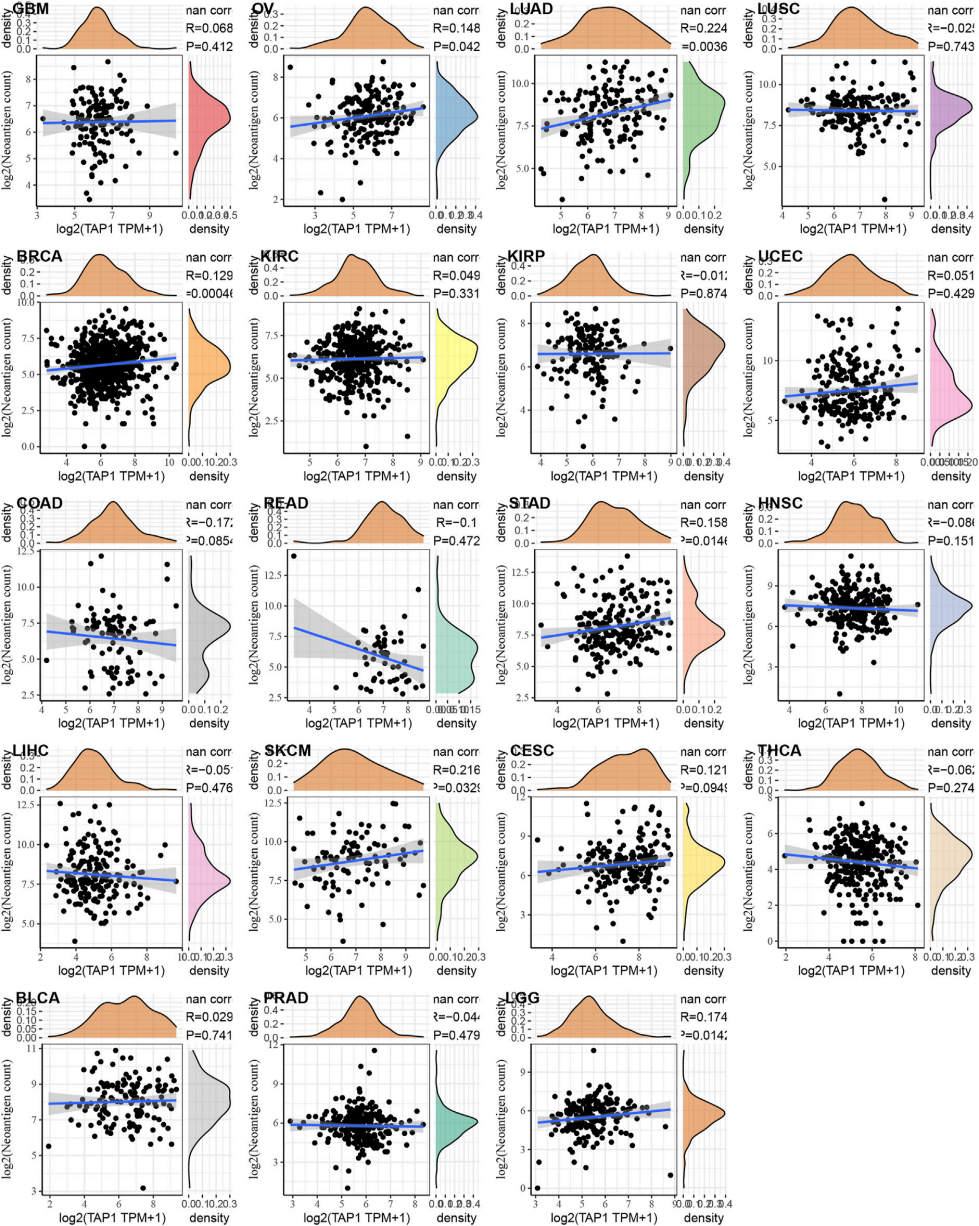
The relationship between transporter associated with antigen processing 1 (TAP1) and neoantigen in various cancers. TAP1 was positively related to neoantigens in ovarian cancer (OC), cervical squamous cell carcinoma (CESC), lung adenocarcinoma (LUAD), breast invasive carcinoma (BRCA), skin cutaneous melanoma (SKCM), and brain lower grade glioma (LGG) (*p* < 0.05), whereas it was negatively correlated in colon adenocarcinoma (COAD) and rectum adenocarcinoma (READ).

Neoantigens referred to a series of protein with specific amino acid sequences produced by tumor cells during gene mutation. These proteins attracted autoimmune cells and caused a series of immune responses, which were more beneficial for the efficacy of tumor-targeted therapy. We analyzed the relationship between TAP1 and neoantigens across various cancers as summarized by Rooney [7]. The results showed that TAP1 was positively related to neoantigens in OC, CESC, LUAD, BRCA, SKCM, and LGG (*p* < 0.05), whereas it was negatively correlated in COAD and rectum adenocarcinoma (READ) (Figure 6). DNA methylation was a form of DNA chemical modification that was commonly observed in tumors. DNA methyltransferases prevented changing the DNA sequence while changing the genetic performance. DNMT1, DNMT2, DNMT3A, and DNMT3B were 4 important methyltransferases, and we analyzed the correlation between TAP1 and these methyltransferases in 33 tumors. TAP1 was positively correlated with DMNT1 in OC, CESC, PAAD, pheochromocytoma and paraganglioma; (PCPG), prostate adenocarcinoma (PRAD), STAD, UVM, BLCA, BRCA, COAD, DLBC, HNSC, kidney chromophobe (KICH), KIRC, KIRP, LAML, LGG, and LIHC (*p* < 0.05). TAP1 was positively related to DMNT2 in PCPG, READ, STAD, TGCT, BLCA, BRCA, DLBC, KICH, KIRC, KIRP, LGG, and LIHC (*p* < 0.05), while it was negatively correlated in OC, CESC, TGCT, and THCA (*p* < 0.05). The remaining results were shown in Supplementary Figure S7.

### Overexpression of TAP1 in OC Patients With Poor Prognosis

**FIGURE 7:**
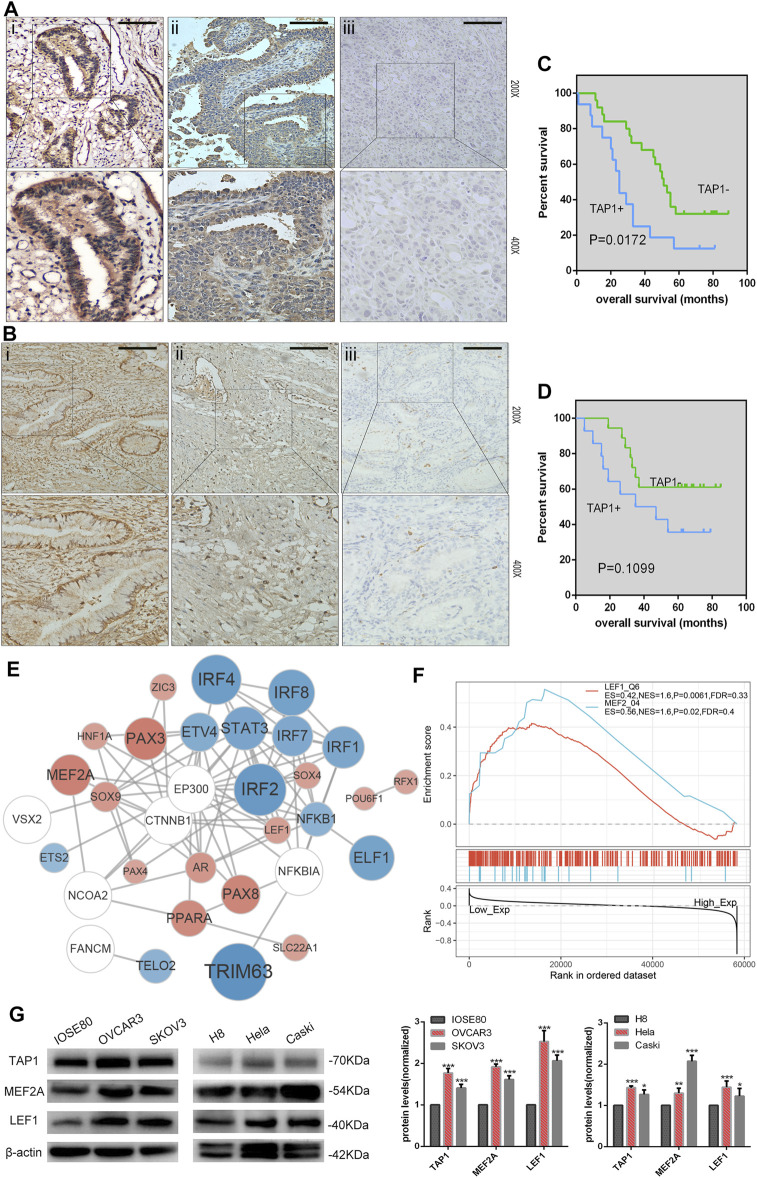
Correlation of transporter associated with antigen processing 1 (TAP1) expression with the prognosis of ovarian cancer (OC) and cervical cancer (CC). **(A)** Immunohistochemical (IHC) analysis of transporter associated with antigen processing 1 (TAP1) protein expression in OC tissues. Original magnifications:×200 and ×400. i represents strong positive staining, ii represents moderately positive staining, and iii represents weakly positive staining. **(B)** IHC analysis of TAP1 protein expression in CC tissues. Original magnifications:×200 and ×400. i represents strong positive staining, ii represents moderately positive staining, and iii represents weakly positive staining. Scale bars 200 μm. **(C)** 5-Year Kaplan–Meier analysis of overall survival in OC patients on the basis of TAP1 protein expression. **(D)** 5-Year Kaplan–Meier analysis of overall survival in CC patients on the basis of the TAP1 protein expression. **(E)** The protein-protein interaction (PPI) network of TAP1 and top 26 correlated transcription factors (TFs) were identified using STRING (https://string-db.org/cgi/input.pl). Color blue represented TFs negatively correlated with TAP1, color red represented TFs positively correlated with TAP1, and color white represented TFs significantly correlated with the top 26 TFs in enrichment analysis. **(F)** Gene set enrichment analysis of transporter associated with antigen processing 1 (TAP1) and pivotal TFs in OC. **(G)** TAP1, MEF2A, LEF1, and β-actin protein expression in normal cell lines and cancer cell lines. Error bars represent the SD of triplicate measurements. *p < 0.05; **p < 0.01; ***p < 0.001.

On the basis of previous results, we speculated that TAP1 may play a role in predicting the prognosis of patients with OC. Additionally, we wanted to understand the potential role of TAP1 in CC, which was another common gynecological cancer. Therefore, we analyzed the expression of TAP1 in clinical samples obtained from patients with OC and CC. We collected 41 OC tissue samples and 32 CC tissue samples and performed immunohistochemical staining to determine the expression of TAP1. Moderate or strong staining, indicating TAP1 expression, was observed in 39.02% (16/41) of OC specimens, whereas 60.98% (25/41) of the samples showed weak staining, indicating negative TAP1 expression. The representative images of OC samples with strong, moderate, or weak TAP1 expression were shown in Figure 7A. Further, we classified the 41 OC specimens into 2 groups according to negative or positive TAP1 expression. The relationship between OS of patients with OC and TAP1 was analyzed using KM method; results of this analysis showed that patients with positive expression of TAP1 had lower 5-years OS than those with negative expression (*p* = 0.0172) (Figure 7C). In CC tissues, 43.75% (14/32) of the samples showed moderate or strong staining, indicating positive TAP1 expression and 56.23% (18/32) of the samples showed weak or negative staining, indicating negative TAP1 expression. The representative images of CC samples were shown in Figure 7B. The KM analysis showed no significant difference between the OS of CC patients and TAP1 expression (*p* = 0.1099) (Figure 7D).

### GSEA of TAP1 in OC

GSEA was used to further investigate the function of TAP1 in OC. We determined the relationship between TAP1 and pivotal TFs and selected the TFs with high normal enrichment score (NES). Then, all the TFs with high NES were added to combine with chip-seq in the chipbase database (http://rna.sysu.edu.cn/chipbase/), and the network diagram (Figure 7E). We found that some well-known TFs associated with OC could probably regulate TAP1 directly, such as signal transducer and activator of transcription 3 (STAT3) and paired box 3 PAX3 (Han et al., 2019; Liang et al., 2020). However, some TFs did not play a role in OC such as myocyte specific enhancer factor 2A (MEF2A) and lymphoid enhancer-binding factor 1 (LEF1) (Figure 7F). Then, we used western blotting to determine the expression of TAP1, MEF2A, and LEF1 in OC cell lines (SKOV3 and OVCAR3) and compared it with that in normal ovarian epithelial cell IOSE80. Further, we analyzed the expression of TAP1, MEF2A, and LEF1 in CC cell lines (Hela and Caski) and normal cervical cell line H8 (Figure 7G). Our results showed that the expression levels of these 3 genes were higher in SKOV3 and OVCAR3 cell lines than in IOSE80 and in Caski and Hela cell lines than in H8. Our results showed that the expression levels of TAP1, MEF2A, and LEF1 were similar in OC and CC.

### Downregulation of TAP1 Reduces Invasion and Migration in OC Cells

**FIGURE 8:**
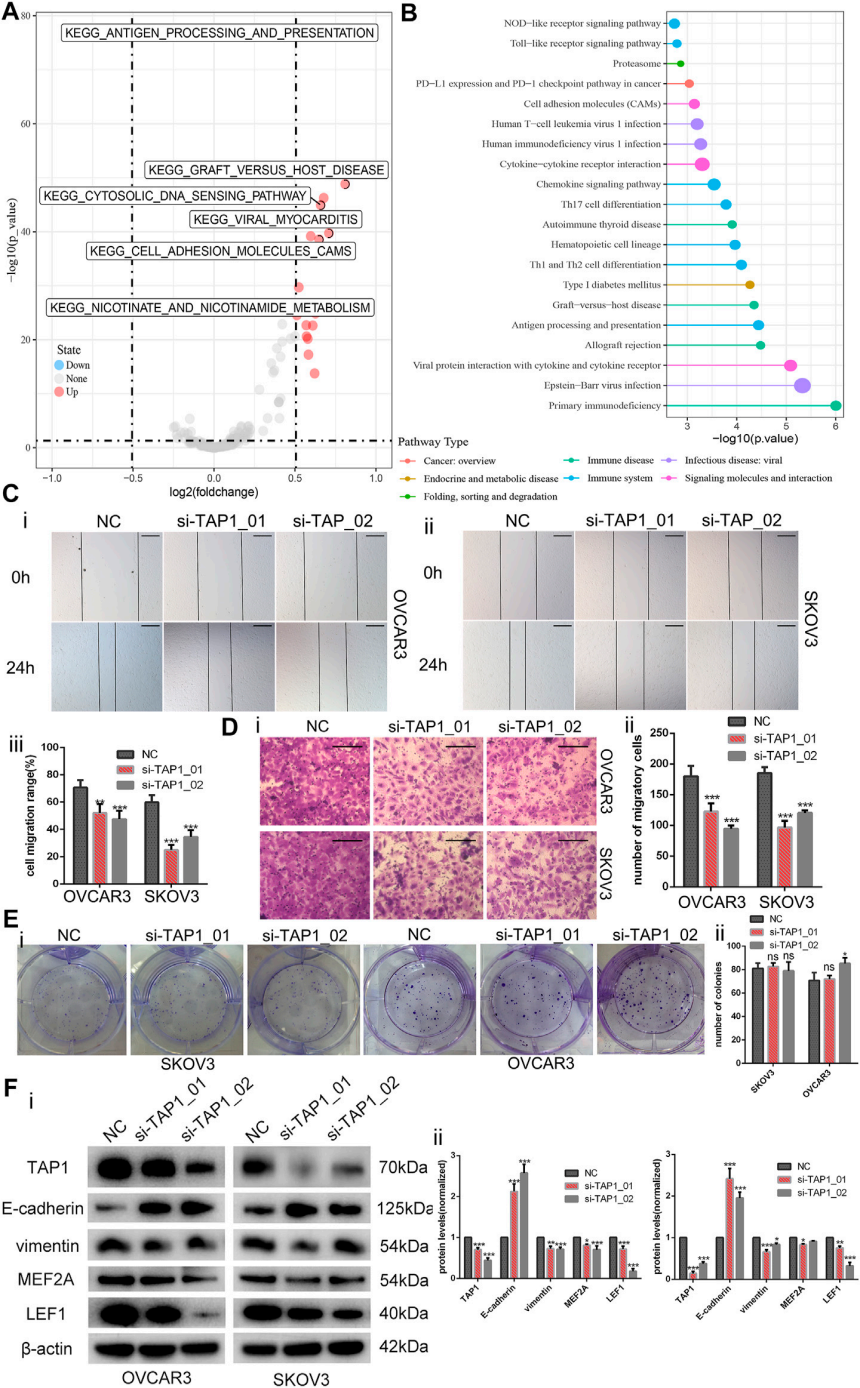
Downregulation of transporter associated with antigen processing 1 (TAP1) reduces the invasion and migration in ovarian cancer cells. **(A)** Then volcano map of TAP1-related pathways by gene set variation analysis. **(B)** Enriching pathway analysis in ovarian cancer of TAP1. **(C)** The migration abilities of OVCAR3 and SKOV3 were photographed (i, ii) and measured (iii) by examining the wound closure after TAP1 knockdown using wound healing assays. Original magnifications, ×100. Scale bars 150 μm. **(D)** Transwell assays were photographed (i) and measured (ii) to detect the migration abilities after TAP1 knockdown in SKOV3 and OVCAR3 cells. Original magnifications, ×200. Scale bars 100 μm. **(E)** Colony formation assays were photographed (i) and measured (ii) to detect the proliferation abilities after TAP1 knockdown in SKOV3 and OVCAR3 cells. **(F)** Effects of TAP1 knockdown on migration-associated protein (E-cadherin and vimentin) and transcription factor (MEF2A and LEF1) were analyzed by western blotting in SKOV3 and OVCAR3 cells (i). Error bars of histogram (ii) represented the SD of triplicate measurements. *p < 0.05; **p < 0.01; ***p < 0.001.

Through KEGG pathway analysis by using GSEA according to TAP1 expression in OC specimens from the TCGA database, we found that TAP1 could influence the cell adhesion molecules pathway, apoptosis pathway, JAK-STAT signaling pathway, and epithelial cell signaling (Supplementary Figure S8). We performed GSVA on all survival-related genes in TCGA OC specimens. On the basis of the expression of TAP1, we then analyzed the different pathways to find that TAP1 could promote the cell adhesion molecules pathway and illustrated through the volcano map (Figure 8A). Further, comparison of the differences in survival-related genes in the TAP1 high and low expression groups and enriching the pathways of differential genes showed that TAP1 could promote the cell adhesion molecules pathway (Figure 8B).

**FIGURE 9:**
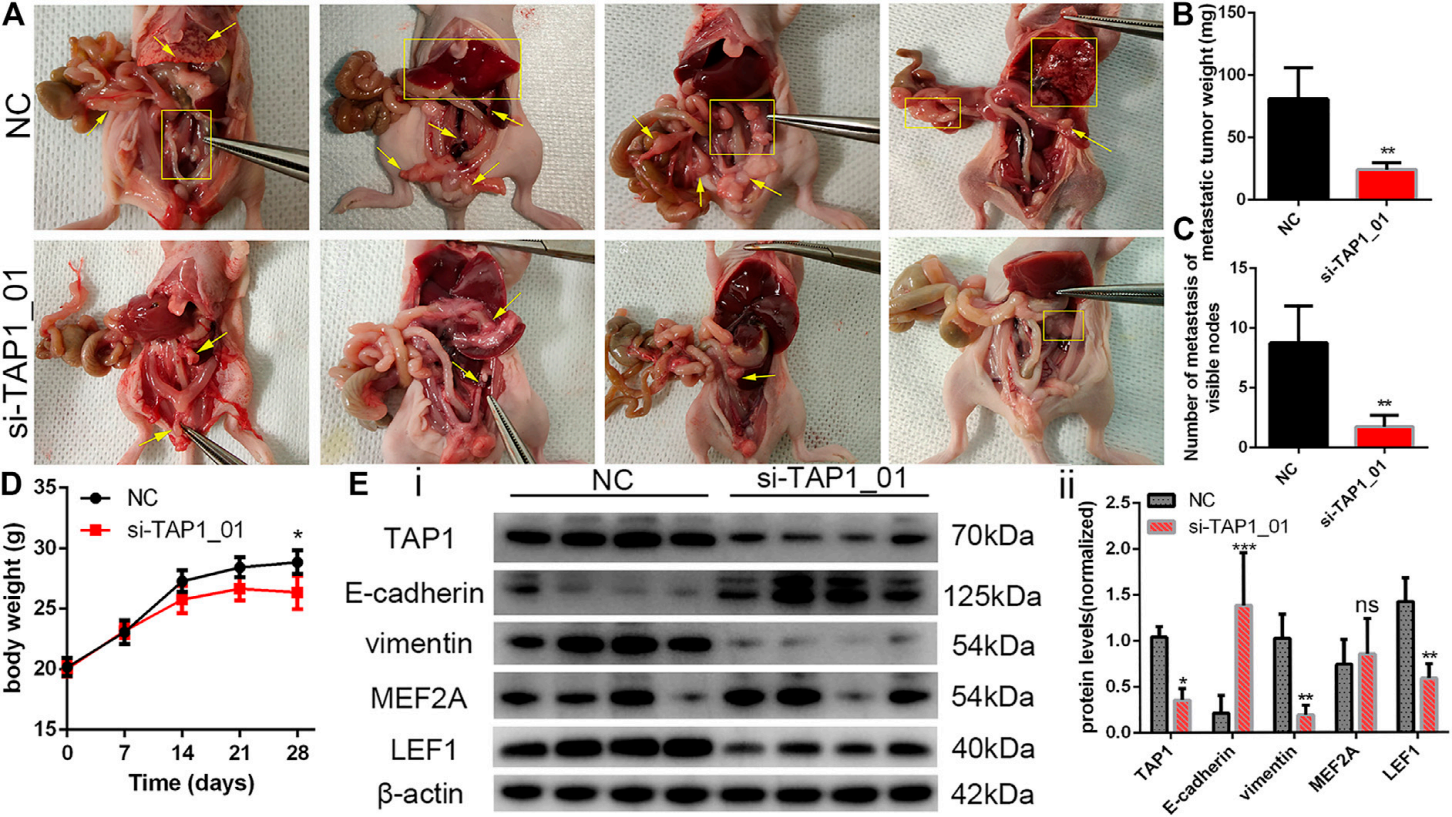
To establish the model of tumor metastasis, SKOV3 cells were injected into nude mice by the i.p. route and randomly treated with si-TAP1_01 or si-NC (*n* = 4/group). **(A)** Representative images of metastatic tumors in the peritoneal cavity. Yellow arrows and rectangles highlighted visible tumor nodes and liver metastasis. **(B,C)** Number of metastasis and total weight of all metastases were shown (mean ± SEM). **(D)** Body weight growth of all mice were shown (mean ± SEM). **(E)** The protein expression of migration-associated protein (E-cadherin and vimentin) and transcription factor (MEF2A and LEF1) in the tumor of si-TAP1_01 group and si-NC group (i). Error bars of histogram (ii) represented the SD of quadruplicate measurements. *p < 0.05; **p < 0.01; ***p < 0.001.

On the basis of the above findings, we wanted to further explore the potential role of TAP1 in the metastasis and migration of OC cells. Thus, we designed 2 different siRNA products (si-TAP1_01 and si-TAP1_02) and a scrambled siRNA as the negative control (NC). The siRNA products were transfected into OVCAR3 and SKOV3 cells, and western blot analysis was performed. The results showed that both si-TAP1 could effectively knock down TAP1 expression. Then, we studied the impact of TAP1 on cell migration and invasion using cell scratch-wound healing assays and transwell cell migration assays. Scratch-wound healing assays revealed that silencing TAP1 in OVCAR3 and SKOV3 cells decreased the wound healing ability (Figure 8C). The results of transwell cell migration assays demonstrated that knockdown of TAP1 significantly reduced the number of cells on membrane filters compared with controls (Figure 8D). Further, we measured the protein levels of cancer cell metastasis-related markers (E-cadherin and vimentin) and the genes of interest (MEF2A and LEF1) were measured. The results showed that TAP1 deficiency decreased vimentin, MEF2A, and LEF1 levels, whereas the levels of E-cadherin protein increased in OVCAR3 and SKOV3 cells (Figure 8F). Through cell proliferation assays (Supplementary Figure S9) and colony formation assays (Figure 8E), we found that TAP1 had no significant effect on the proliferation of ovarian cancer cells. Thus we eliminated the potential effect of proliferation on the aforementioned scratch-wound healing and transwell assays. To further confirm the metastasis-promoting effects of TAP1, we performed *in vivo* xenograft tumor experiments in nude mice. Accordingly, we injected SKOV3 cells into nude mice intraperitoneally and treated them with TAP1 siRNA (si-TAP1_01) or scrambled NC RNA (si-NC). The results demonstrated that the number and weight of metastatic tumors in peritoneal cavity were lower in the si-TAP1_01 group than in the si-NC group (Figures 9A–C), while the weight growth rates were higher in the si-NC group (Figure 9D). Higher level of TAP1 in metastatic tumors were associated with higher levels of vimentin and LEF1, while associated with lower level of E-cadherin. However the enhancement of TAP1 *in vivo* did not seem to notably effect the expression of MEF2A (Figure 9E). These results demonstrated that TAP1 could play a role in the invasion and metastasis of OC cells and TAP1 may regulate the transcription factor LEF1 in OC. Further studies were necessary to confirm these findings.

## Discussion

TAP includes TAP1 and TAP2, which belong to the ABC transporter superfamily and consist of 2 transmembrane domains, consisting of a substrate binding site and 2 nucleotide binding domains (Praest et al., 2018). TAP1 and TAP2 are responsible for transporting antigen peptides from the cytoplasm to the lumen of the endoplasmic reticulum, then loading them on the MHC class I molecules of the MHC. TAP1 and TAP2 can provide endogenous protein peptides for CD8^+^ cytotoxic T cells, so the TAP family plays a role in maintaining the normal function of the immune system (Meng et al., 2018).

Recent studies showed that TAP1 was closely related to a variety of tumors, which may be related to tumor cells evading the recognition of cytotoxic T cells by shutting down peptide delivery. Chow et al. found that increased copy number of TAP1 was closely related to histological duct variation in prostate cancer (Chow et al., 2021). TAP1 was involved in IL-27-mediated anti-PD-1 antibody immunotherapy in small cell lung cancer (Carbotti et al., 2017). The level of TAP1 could affect the survival time of patients with oral squamous cell carcinoma (SCCOT) and breast cancer, and may serve as a tumor marker for the early diagnosis and follow-up of SCCOT (Henle et al., 2017; Attaran et al., 2020). Seliger et al. reported that miR-200a-5p reduced HLA-I expression on the membrane of melanoma tumor cells by downregulating the level of TAP1, causing the tumor cells to escape the immune system and reducing the survival time of tumor patients (Lazaridou et al., 2020). In this study, we analyzed the data derived from TCGA database and GTEx database and found that TAP1 expression was elevated in lung cancer, breast cancer, pancreatic cancer, renal cell carcinoma, and other tumors and was correlated with the survival state of patients, which was consistent with the findings reported previously (Xu et al., 2013; Xu et al., 2019). Moreover, our results showed that TAP1 expression levels were increased in gynecological tumors, including OC, CC, and endometrial cancer. Our results showed that TAP1 was significantly correlated with the prognosis of patients with OC, including data in TCGA and clinical specimens. The results indicated that TAP1 could function as a prognostic marker for OC.

TAP1 plays a role in tumor development and resistance to treatment mainly by affecting tumor immune infiltration. Marcoto et al. found that the levels of TAP1 and CD80 decreased in breast cancer stem cells after decitabine treatment, thereby reducing the recognition and killing effect of T cells and promoting tumor cell growth (Sultan et al., 2018). Ling et al. confirmed that TAP1 in colorectal cancer was positively related to TIICs, including CD3^+^ T cells and FOXP3+ T cells (Ling et al., 2017). Loss of TAP1 expression in melanoma was correlated with the increase of Treg cells and neutrophils in cancer, which could change the immune microenvironment and participate in the reversal of resistance to anti-PD1 therapy (Zhang et al., 2021). The results of analysis of the TIMER database showed a relationship between TAP1 and the infiltration of a variety of TIICs, including CD4^+^ T cells, CD8^+^ T cells, B cells, neutrophils, dendritic cells, and macrophages. The results showed that TAP1 was related to all of these 6 immune cells in KIRC, COAD, BRCA, and LUSC. Further, we analyzed the correlation between TAP1, IMMUNE score, STROMAL score, and ESTIMATE score in a variety of tumors. Then, we identified the top 3 tumors—BRCA, COAD, and KIRC. We directly explained the significant correlation between TAP1 and tumor immune cell infiltration in OC as well as immune scores, which suggested that TAP1 could influence the abundance of TIICs in OC.

OC is one of the most common causes of death in women with cancers, with more than 120,000 deaths worldwide every year (Vaughan et al., 2011). Approximately 70% of women with OC are in the late stage during the initial diagnosis, while 5-years survival rate is less than 50% (Torre et al., 2018). Immunotherapy includes anti-PDL-1 therapy and PARP inhibitor therapy has shown promising results in the treatment of OC; however, many patients may relapse after receiving first-line treatment. The therapeutic effect is related to the tumor heterogeneity and its surrounding tumor microenvironment (Baci et al., 2020). Thus, it is very important to find effective prognostic markers of OC and related genes of immune regulation. Based on the aforementioned research, we analyzed the correlation between TAP1 and 47 recognized immune checkpoint genes in pan-cancers through Spearman correlation test. We found that TAP1 was significantly correlated with genes such as CD274 (PDL-1), CTLA4, CD244, and LAG3 in OC, in which the above genes were all related to the occurrence and development and the curative effect of existing immunotherapy (Huang et al., 2015; Macgregor et al., 2019; Marth et al., 2019). Chen et al. found that the degree of methylation of TAP1 was correlated with the degree of CD8^+^ T cell infiltration, while the reduction of CD8^+^ T cells infiltration was related to the recurrence of high-grade serous epithelial OC (Wang et al., 2014). By analyzing the correlation between TAP1 and methyltransferases in pan-cancers, we found that TAP1 had a significant correlation with the 4 methyltransferases in KICH and DLBC. In OC, TAP1 had a significant correlation with DNMT1, which was involved in the metastasis and cisplatin resistance of OC (Cao et al., 2017). Therefore, we speculated that DNMT1 changed the methylation status of TAP1 and affected the infiltration of tumor immune cells thereby changing the prognosis of OC, which was worthy of further verification.

In order to further analyze the relationship between TAP1 and OC, we performed GSEA. The results of GSEA showed that TAP1 was associated with the apoptosis signaling, JAK/STAT signaling, TOLL-like receptor signaling and cell adhesion signaling in OC, which indicated that TAP1 may affect the formation of tumor, proliferation, and migration in OC in addition to immune-related pathways. Analysis of transcription factors in GSEA showed that some core TFs may have a relation with TAP1 in OC, including ETS transcription factor (ELK1), interferon regulatory factor (IRF), STAT3, and MEF2A, which affected metastasis, cisplatin resistance, and apoptosis in OC [(Li et al., 2019), (Praest et al., 2018), (Gu et al., 2021), (Pavan et al., 2013)]. We found that MEF2A and LEF1 may participate in OC and regulate TAP1. MEF2A could promote the proliferation and metastasis of tumor cells by inducing epithelial-mesenchymal transition (EMT) and activating the WNT/β-catenin signaling pathway (Xiao et al., 2021). LEF1 could promote tumor cell metastasis by participating in EMT and regulating the intracellular reactive oxygen species (ROS). These results provided a research basis for our follow-up study of the mechanism of TAP1 in OC. However, further studies are required to understand the function of TAP1 in OC.

Our results showed that the expression of TAP1 was increased in many tumors. High expression level of TAP1 was positively related to the poor prognosis of various tumors and immune cell infiltration. Additionally, we found that TAP1 affected the development of OC in different ways. According to the analysis of tumor neoantigen load and immune checkpoint, TAP1 had the potential to become a novel tumor-targeted drug. Thus, TAP1 may be a new therapeutic target for OC and other kinds of cancers. Thus, our results showed that TAP1 could function as a biomarker for cancer diagnosis and prognosis.

## Data Availability

The data that support the findings of this study are openly available in the Cancer Genome Atlas (TCGA) database at https://www.cancer.gov/t-cga, the Genotype-Tissue Expression (GTEx) database at https://www.gtexportal.org/home/, the Cancer Cell Line Encyclopedia (CCLE) database at https://portals.broadinstitute.org/ccle, and the TIMER database at http://timer.cistrome.org/. The data that support the research about cancer neoantigens are openly available in “Molecular and genetic properties of tumors associated with local immune cytolytic activity“ at https://pubmed.ncbi.nlm.nih.gov/25-594174/.

## Glossary

ACC: adrenocortical carcinoma
BLCA: bladder urothelial carcinoma
BRCA: breast invasive carcinoma
CESC: cervical squamous cell carcinoma and endocervical adenocarcinoma
CHOL: cholangiocarcinoma
COAD: colon adenocarcinoma
READ: rectum adenocarcinoma esophageal carcinoma: rectum adenocarcinoma
DLBC: lymphoid neoplasm diffuse large B-cell lymphoma
ESCA: esophageal carcinoma
GBM: glioblastoma multiforme
HNSC: head and neck squamous cell carcinoma
KICH: kidney chromophobe
KIPAN: pan-kidney cohort (KICH + KIRC + KIRP)
KIRC: kidney renal clear cell carcinoma
KIRP: kidney renal papillary cell carcinoma
LAML: acute myeloid leukemia
LGG: brain lower grade glioma
LIHC: liver hepatocellular carcinoma
LUAD: lung adenocarcinoma
LUSC: lung squamous cell carcinoma
MESO: mesothelioma
OC: ovarian serous cystadenocarcinoma
PAAD: pancreatic adenocarcinoma
PCPG: pheochromocytoma and paraganglioma
PRAD: prostate adenocarcinoma
READ: rectum adenocarcinoma esophageal carcinoma: rectum adenocarcinoma
SARC: sarcoma
SKCM: skin cutaneous melanoma
STAD: stomach adenocarcinoma
STES: stomach and esophageal carcinoma
TGCT: testicular germ cell tumors
THCA: thyroid carcinoma
THYM: thymoma
UCEC: uterine corpus endometrial carcinoma
UCS: uterine carcinosarcoma
UVM: uveal melanoma
