# Corrigendum: Comprehensive analysis of the potential immune-related biomarker transporter associated with antigen processing 1 that inhibits metastasis and invasion of ovarian cancer cells

**DOI:** 10.3389/fmolb.2022.984209

**Published:** 2022-09-02

**Authors:** Xiaoxue Li, Shiyu Zeng, Yiling Ding, Yanting Nie, Mengyuan Yang

**Affiliations:** Department of Obstetrics and Gynecology, The Second Xiangya Hospital of Central South University, Changsha, China

**Keywords:** pan-cancer, ovarian cancer, cervical cancer, transporter associated with antigen processing 1, tumor-infiltrating immune cells, immune checkpoint, metastasis

In the published article, there was an error in “Figure 8D” as published. The picture in Figure 8D was wrong when we uploaded the pictures. The corrected Figure 8 and its caption “Downregulation of transporter associated with antigen processing 1 (TAP1) reduces the invasion and migration in ovarian cancer cells” appear below.

**FIGURE 8:**
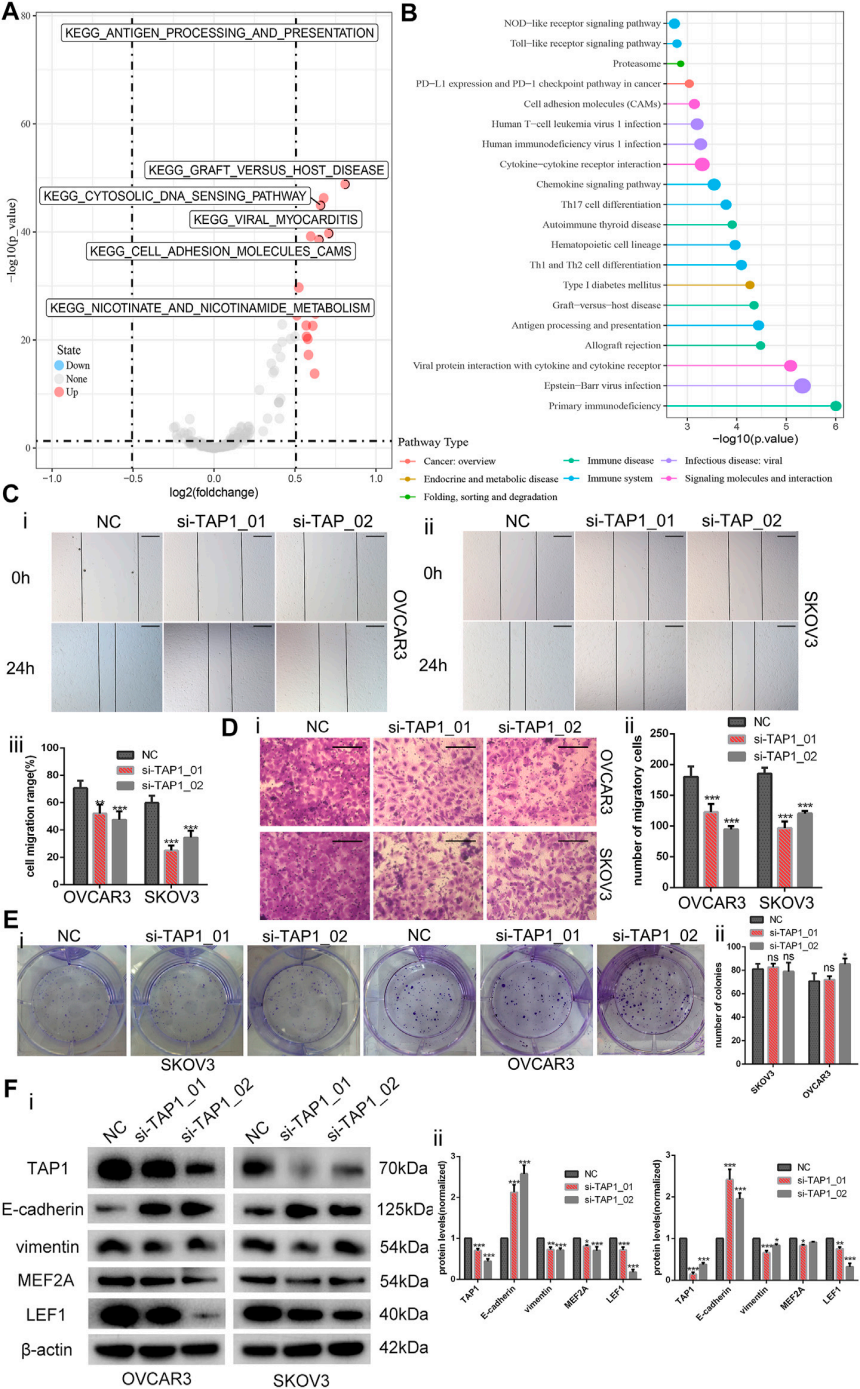
Downregulation of transporter associated with antigen processing 1 (TAP1) reduces the invasion and migration in ovarian cancer cells. **(A)** The volcano map of TAP1-related pathways by gene set variation analysis. **(B)** Enriching pathway analysis in ovarian cancer of TAP1. **(C)** The migration abilities of OVCAR3 and SKOV3 were photographed (i, ii) and measured (iii) by examining the wound closure after TAP1 knockdown using wound healing assays. Original magnifications, ×100. Scale bars 150 μm. **(D)** Transwell assays were photographed (i) and measured (ii) to detect the migration abilities after TAP1 knockdown in SKOV3 and OVCAR3 cells. Original magnifications, ×200. Scale bars 100 μm. **(E)** Colony formation assays were photographed (i) and measured (ii) to detect the proliferation abilities after TAP1 knockdown in SKOV3 and OVCAR3 cells. **(F)** Effects of TAP1 knockdown on migration-associated protein (E-cadherin and vimentin) and transcription factor (MEF2A and LEF1) were analyzed by western blotting in SKOV3 and OVCAR3 cells (i). Error bars of histogram (ii) represented the SD of triplicate measurements. *p < 0.05; **p < 0.01; ***p < 0.001.

The authors apologize for this error and state that this does not change the scientific conclusions of the article in any way. The original article has been updated.

