# Supplementary figures and images for: Comprehensive Analysis of the Potential Immune-Related Biomarker Transporter Associated With Antigen Processing 1 That Inhibits Metastasis and Invasion of Ovarian Cancer Cells
Source: Front Mol Biosci. 2021 Dec 10;8:763958. doi: 10.3389/fmolb.2021.763958 (PMC8702961; doi:10.3389/fmolb.2021.763958)

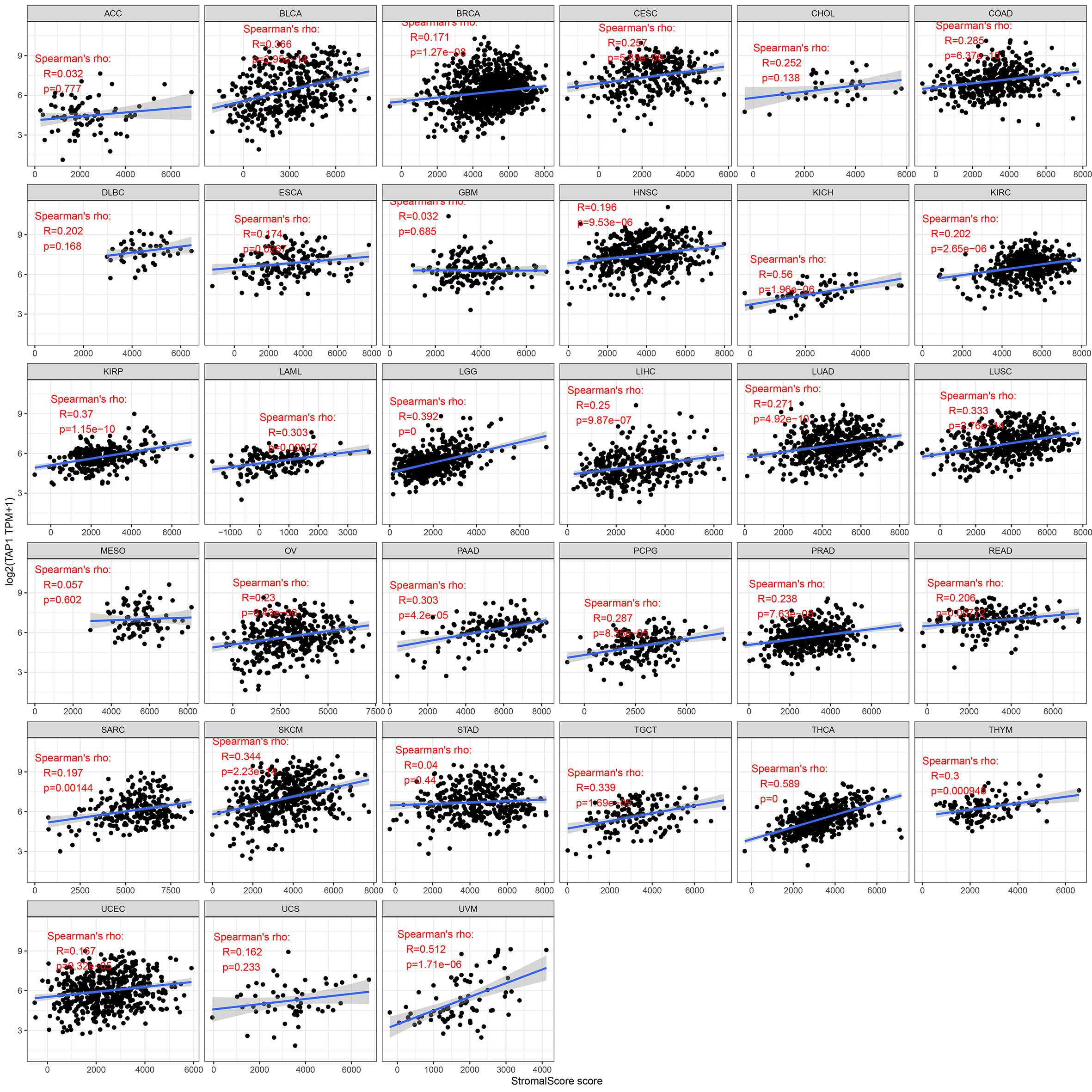

Supplement: Supplementary file 1 [file Image6.TIF]

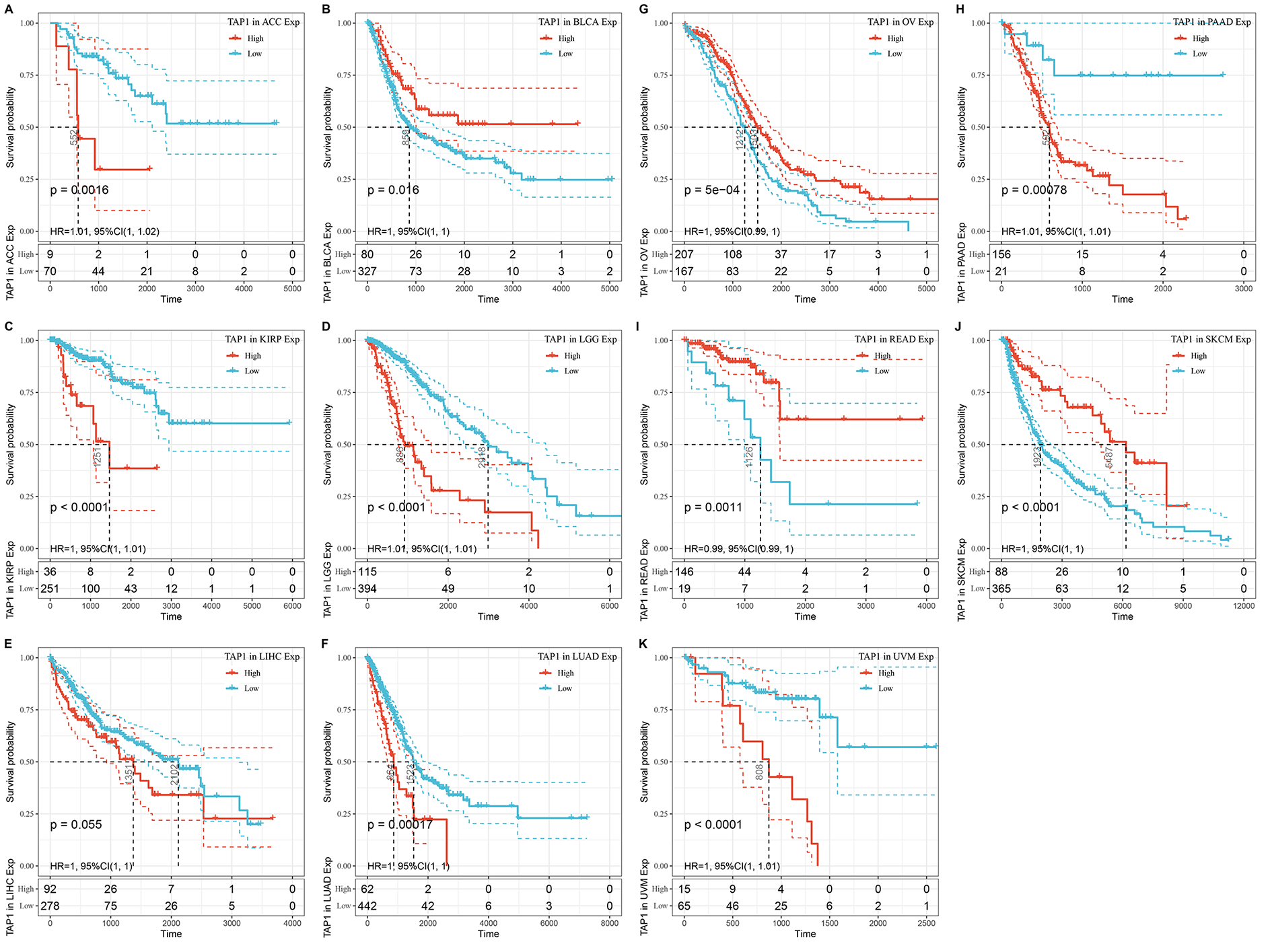

Supplement: Supplementary file 2 [file Image3.TIF]

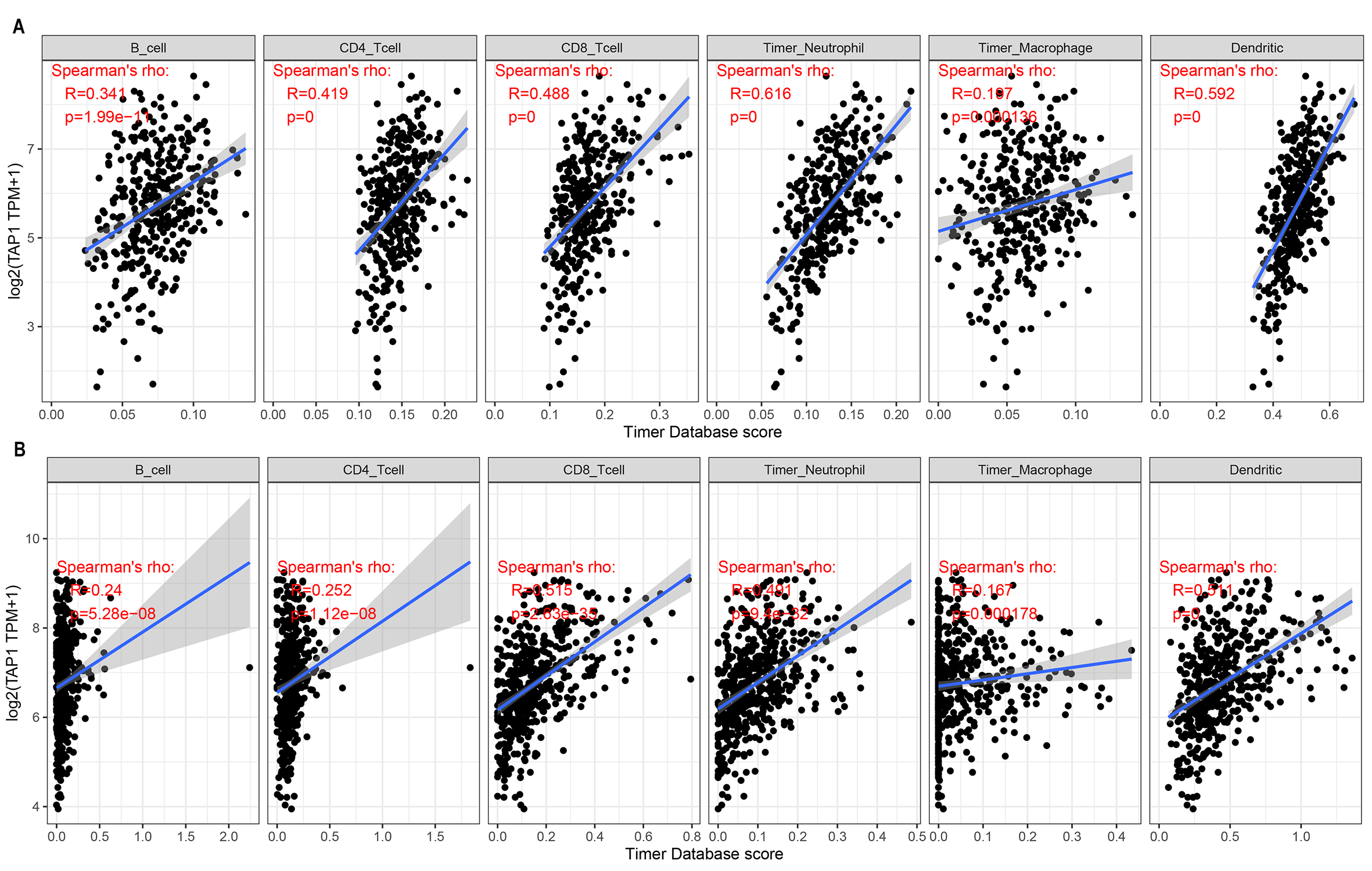

Supplement: Supplementary file 3 [file Image4.TIF]

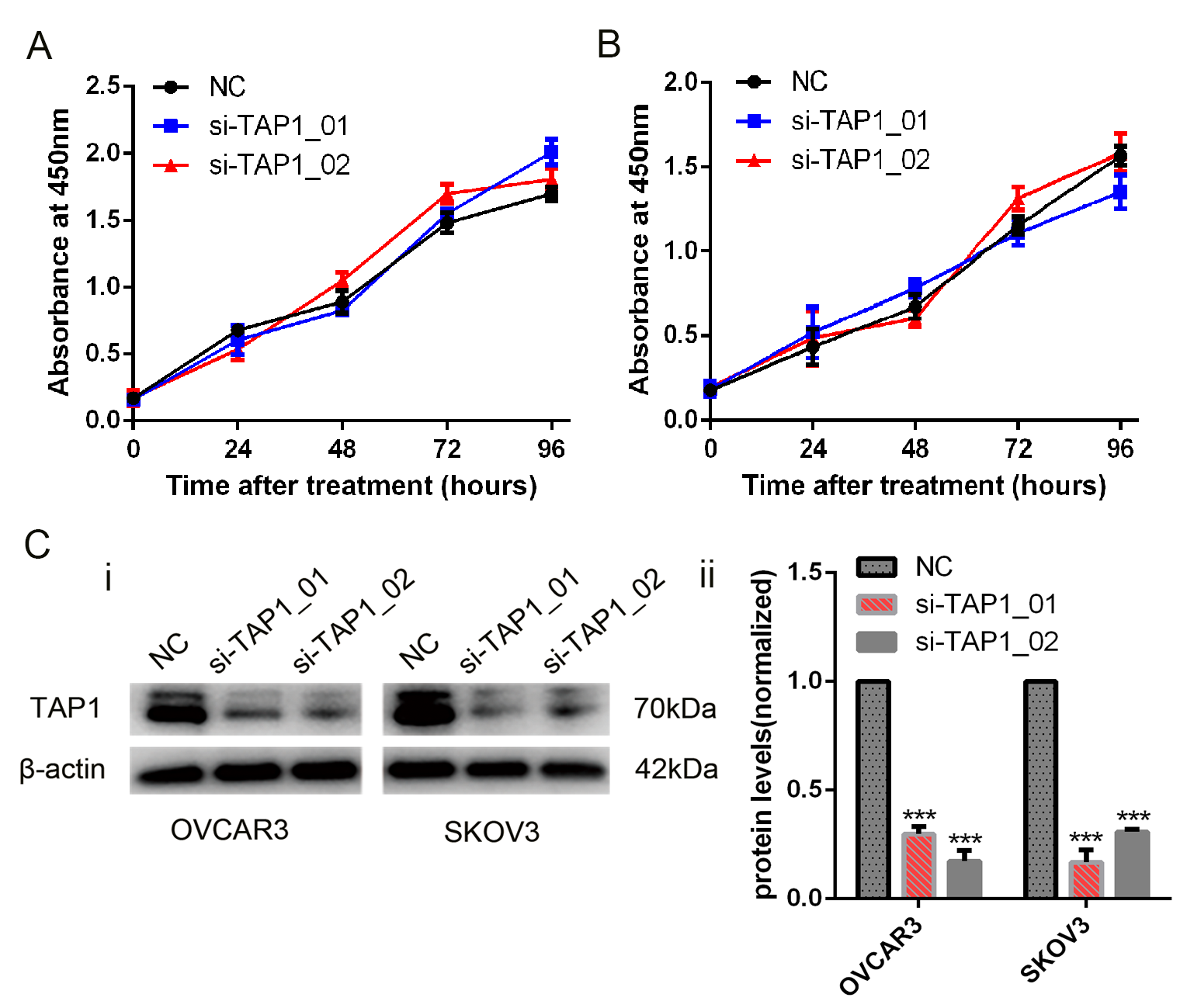

Supplement: Supplementary file 4 [file Image9.TIF]

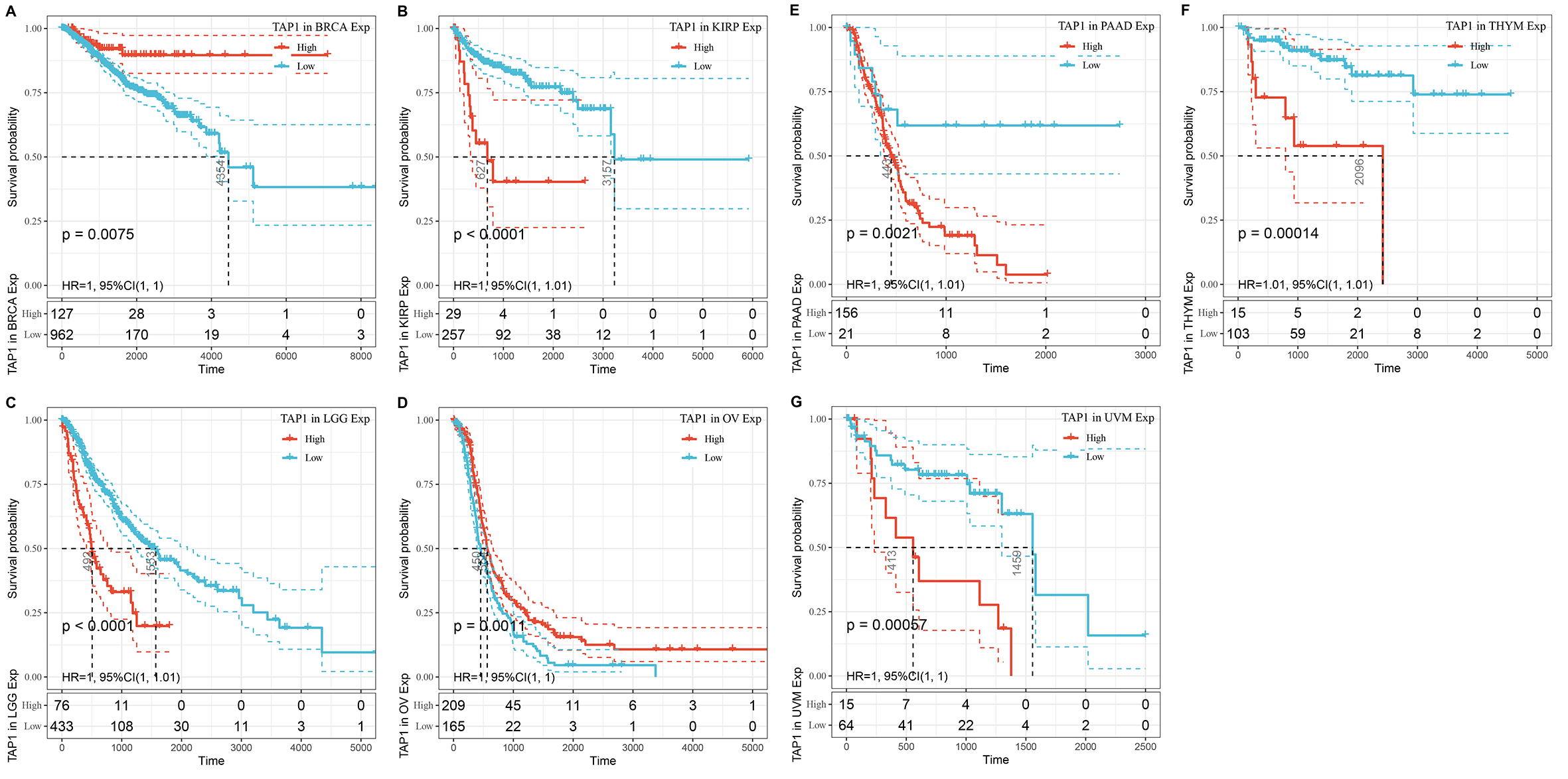

Supplement: Supplementary file 5 [file Image2.TIF]

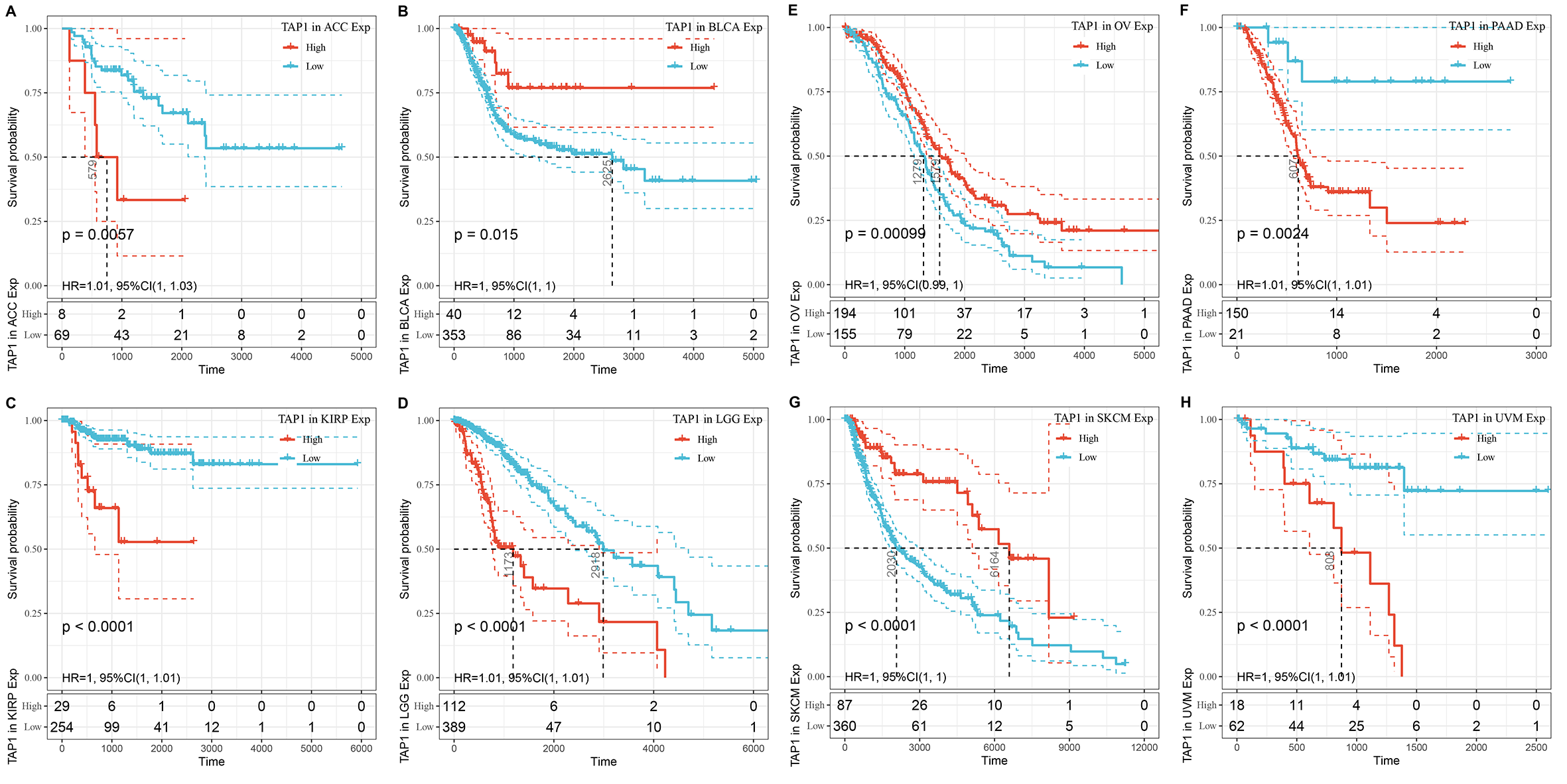

Supplement: Supplementary file 6 [file Image1.TIF]

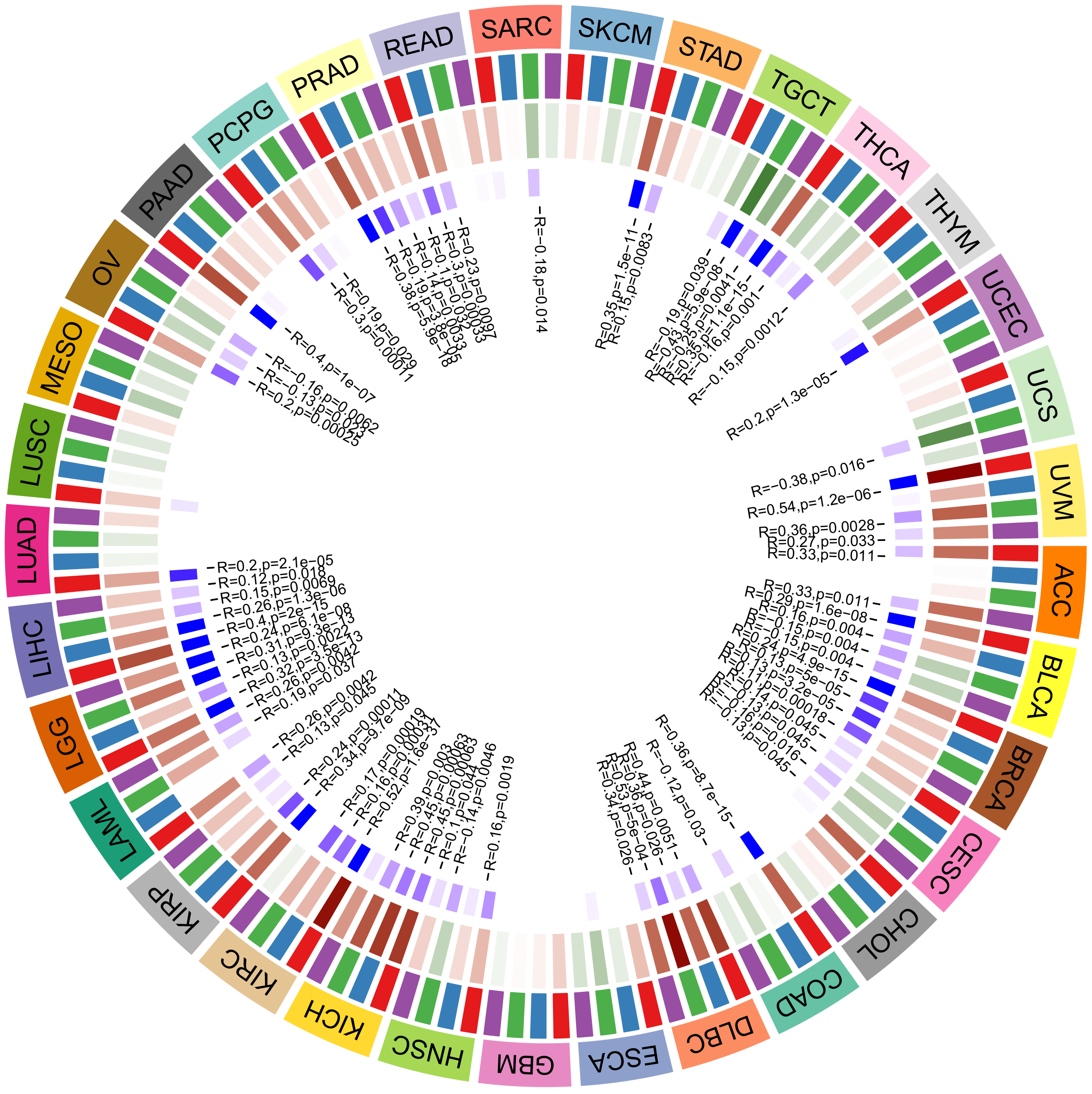

Supplement: Supplementary file 7 [file Image7.TIF]

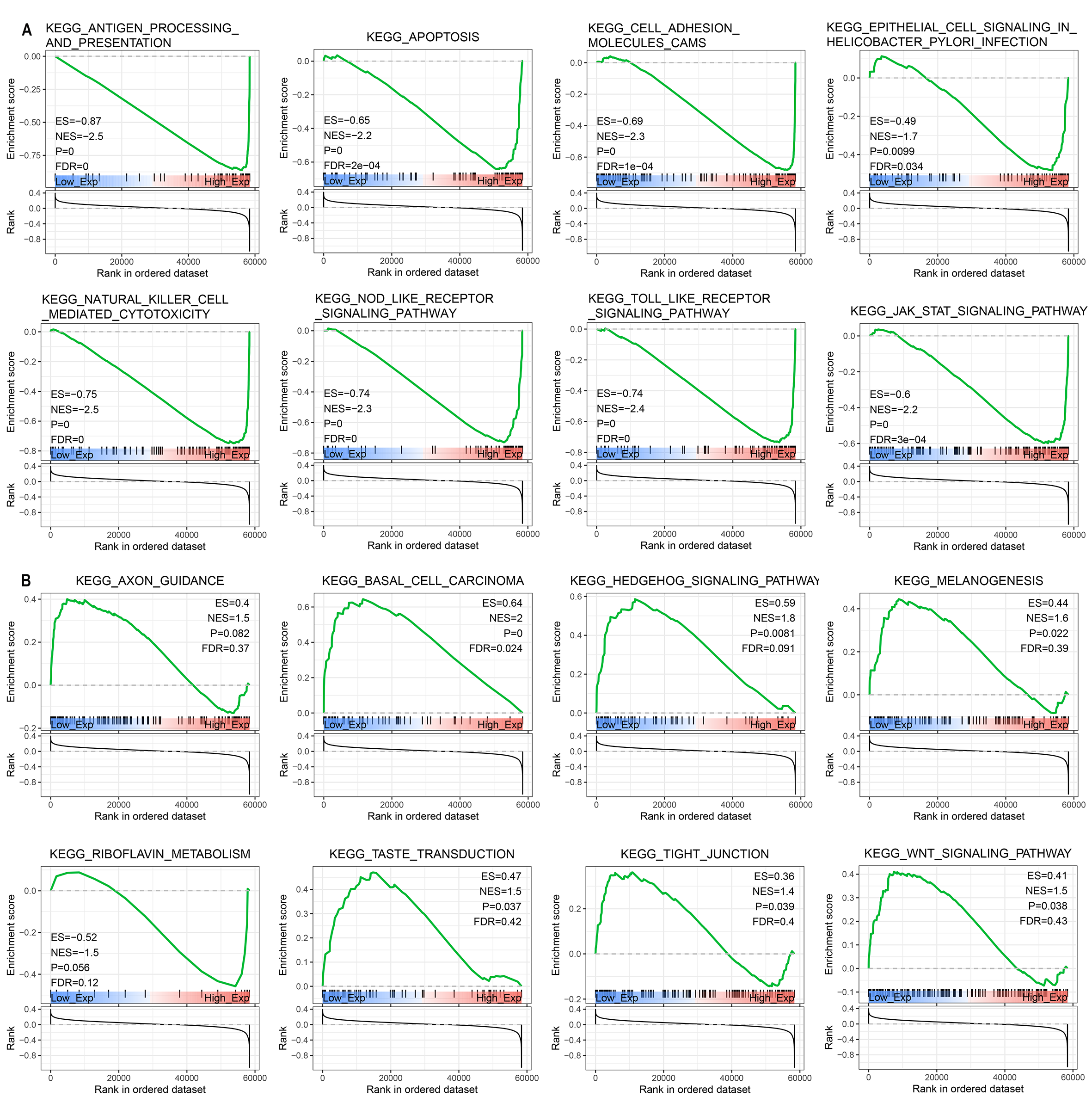

Supplement: Supplementary file 8 [file Image8.TIF]

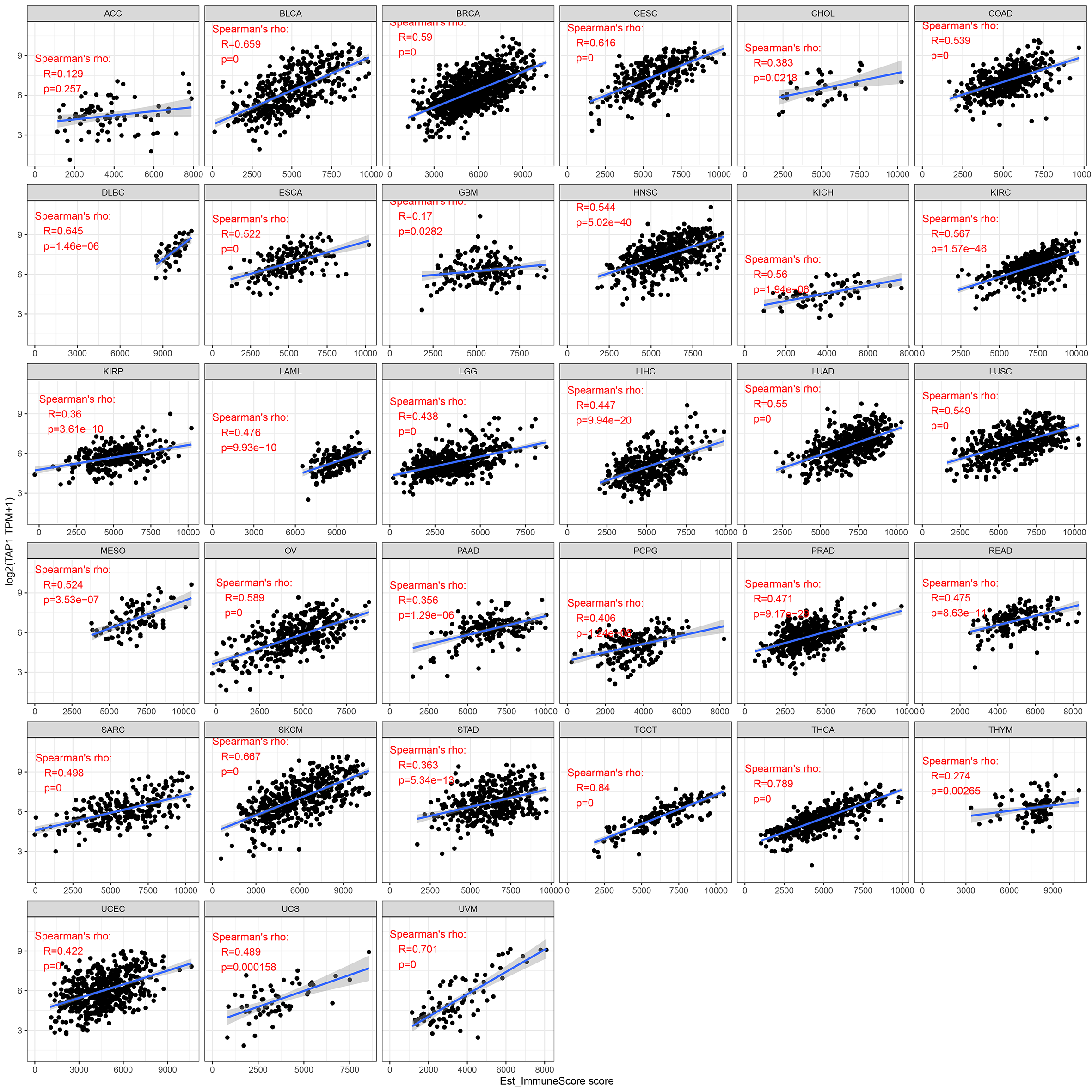

Supplement: Supplementary file 9 [file Image5.TIF]
