# Supplementary material for: Comprehensive Analysis of the Potential Immune-Related Biomarker Transporter Associated With Antigen Processing 1 That Inhibits Metastasis and Invasion of Ovarian Cancer Cells
Source: Front Mol Biosci. 2021 Dec 10;8:763958. doi: 10.3389/fmolb.2021.763958 (PMC8702961; doi:10.3389/fmolb.2021.763958)
Supplement: Supplementary file 10 [file DataSheet1.docx]

**Figure S1**

Kaplan-Meier analysis of disease special survival on the basis of the expression level of TAP1 in pan-cancer.

**Figure S2**

Kaplan-Meier analysis of progression-free survival on the basis of the expression level of TAP1 in pan-cancer.

**Figure S3**

Kaplan-Meier analysis of overall survival on the basis of the expression level of TAP1 in pan-cancer.

**Figure S4**

**(A)** The correlation of TAP1 with 6 TIICs in OC. **(B)** The correlation of TAP1 with 6 TIICs in CESC.

**Figure S5**

The correlation of TAP1 with IMMUNEscore of algorithm in multiple tumors.

**Figure S6**

The correlation of TAP1 with STROMALscore of algorithm in multiple tumors.

**Figure S7**

The relationship between TAP1 expression and 4 methyltransferases. Color red represents DNMT1, color blue represents DNMT2, color green represents DNMT3a, and color purple represents DNMT3b.

**Figure S****8**

KEGG pathway analysis of TAP1 by gene set enrichment analysis in ovarian cancer. **(A)** KEGG pathways negatively correlated with TAP1. **(B)** KEGG pathways positively correlated with TAP1.

**Figure S9**

Effect of TAP1 knockdown on proliferation of OC cells. CCK-8 assays were performed to determine the effects of TAP1 knockdown on the proliferation of OVCAR3 cells **(A)** and SKOV3 cells **(B)**. Cell viability was determined at 0, 24, 48, 72 and 96 h. **(C)** Western blotting assays validated the efficacy of siRNAs knocking down TAP1 (i) and the results were measured (ii). Error bars represented the standard deviation (s.d.) of triplicate measurements. *P < 0.05; **P < 0.01; ***P < 0.001.
